# Retrospective ANalysis of multi-drug resistant Gram-nEgative bacteRia on veno-venous extracorporeal membrane oxygenation. The multicenter RANGER STUDY

**DOI:** 10.1186/s13054-024-05068-x

**Published:** 2024-08-27

**Authors:** Annalisa Boscolo, Andrea Bruni, Marco Giani, Eugenio Garofalo, Nicolò Sella, Tommaso Pettenuzzo, Michela Bombino, Matteo Palcani, Emanuele Rezoagli, Matteo Pozzi, Elena Falcioni, Elisa Pistollato, Eugenio Biamonte, Francesco Murgolo, Graziella D’Arrigo, Mercedes Gori, Giovanni Luigi Tripepi, Leonardo Gottin, Federico Longhini, Salvatore Grasso, Paolo Navalesi, Giuseppe Foti

**Affiliations:** 1 Department of Medicine (DIMED), University of Padua, 13 Gallucci Street, 35121 Padua, Italy; 2 Institute of Anesthesia and Critical Care, Padua University Hospital, Padua, Italy; 3 Department of Cardiac, Thoracic, Vascular Sciences and Public Health, University of Padua, Padua, Italy; 4 Department of Medical and Surgical Sciences, Magna Graecia University, Catanzaro, Italy; 5 School of Medicine and Surgery, University of Milano-Bicocca, Monza, Italy; 6 Department of Emergency and Critical Care, IRCSS San Gerardo Dei Tintori, Monza, Italy; 7 Department of Surgery, Dentistry, Paediatrics and Gynaecology, University of Verona, Verona, Italy; 8 Cardiothoracic and Vascular Intensive Care Unit, Verona University Hospital, Verona, Italy; 9 Department of Precision and Regenerative Medicine and Ionian Area, School of Medicine, University of Bari “Aldo Moro”, Bari, Italy; 10 CNR-IFC, Institute of Clinical Physiology of Reggio Calabria, Reggio Calabria, Italy; 11 CNR-IFC, Institute of Clinical Physiology of Rome, Rome, Italy

**Keywords:** ECMO, ESBL, Extracorporeal membrane oxygenation, Extended-spectrum beta-lactamase, Multi-drug resistant, MDR, MDRO

## Abstract

**Background:**

Veno-venous extracorporeal membrane oxygenation (V-V ECMO) is a rapidly expanding life-support technique worldwide. The most common indications are severe hypoxemia and/or hypercapnia, unresponsive to conventional treatments, primarily in cases of acute respiratory distress syndrome. Concerning potential contraindications, there is no mention of microbiological history, especially related to multi-drug resistant (MDR) bacteria isolated before V-V ECMO placement. Our study aims to investigate: (i) the prevalence and incidence of MDR Gram-negative (GN) bacteria in a cohort of V-V ECMOs; (ii) the risk of 1-year mortality, especially in the case of predetected MDR GN bacteria; and (iii) the impact of annual hospital V-V ECMO volume on the probability of acquiring MDR GN bacteria.

**Methods:**

All consecutive adults admitted to the Intensive Care Units of 5 Italian university-affiliated hospitals and requiring V-V ECMO were screened. Exclusion criteria were age < 18 years, pregnancy, veno-arterial or mixed ECMO-configuration, incomplete records, survival < 24 h after V-V ECMO. A standard protocol of microbiological surveillance was applied and MDR profiles were identified using in vitro susceptibility tests. Cox-proportional hazards models were applied for investigating mortality.

**Results:**

Two hundred and seventy-nine V-V ECMO patients (72% male) were enrolled. The overall MDR GN bacteria percentage was 50%: 21% (n.59) detected before and 29% (n.80) after V-V ECMO placement. The overall 1-year mortality was 42%, with a higher risk observed in predetected patients (aHR 2.14 [1.33–3.47], *p* value 0.002), while not in ‘V-V ECMO-acquired MDR GN bacteria’ group (aHR 1.51 [0.94–2.42], *p* value 0.090), as compared to ‘non-MDR GN bacteria’ group (*reference*). Same findings were found considering only infections. A larger annual hospital V-V ECMO volume was associated with a lower probability of acquiring MDR GN bacteria during V-V ECMO course (aOR 0.91 [0.86–0.97], *p* value 0.002).

**Conclusions:**

21% of MDR GN bacteria were detected before; while 29% after V-V ECMO connection. A history of MDR GN bacteria, isolated before V-V ECMO, was an independent risk factor for mortality. The annual hospital V-V ECMO volume affected the probability of acquiring MDR GN bacteria.

*Trial Registration* ClinicalTrial.gov Registration Number NCTNCT06199141, date 12.26.2023.

**Supplementary Information:**

The online version contains supplementary material available at 10.1186/s13054-024-05068-x.

## Background

Veno-venous extracorporeal membrane oxygenation (V-V ECMO) is a rapidly expanding life-support technique worldwide [1–4. An extracorporeal oxygenator operates in series, completely substituting the patient’s lung physiological gas exchange function 1, 5–8. To date, the Extracorporeal Life Support Organisation (ELSO) registry has recorded more than 56.000 cases of adult respiratory ECMO, mostly due to severe acute respiratory distress syndrome (ARDS) 1. While there are some, internationally accepted, indications for V-V ECMO initiation, identifying potential contraindications for V-V ECMO placement is still a matter of debate 1, 3, 5. In fact, few conditions are considered contraindications due to their association with poor outcome 1. These include mechanical ventilation with non-protective settings for more than 7 days before V-V ECMO placement, recent or expanding central nervous system hemorrhage, advanced age, non-recoverable comorbidities and terminal malignancy 1. To date, pre-existing microbiological history is not included among V-V ECMO contraindications 9. However, a retrospective analysis of the ELSO international registry, among 2.355 adult patients treated by V-V and V-A ECMO, identified infectious complications as an important variable independently associated with poor hospital survival 10. In addition, many authors described as bloodstream infections (BSI), mostly occurring during V-A ECMOs, can impact on ECMO duration, weaning from mechanical ventilation and ICU stay 11, 12. The situation may be even more challenging in the case of isolation of multi-drug resistant (MDR) Gram-negative (GN) pathogens [13–15. Indeed, the isolation of MDR GN bacteria has been shown to be an independent risk of death in several studies enrolling mixed populations in Intensive Care Unit (ICU) [16–19. In patients affected by ARDS and requiring V-V ECMO, data are still lacking about the real incidence of MDR GN bacteria, including extended-spectrum β-lactamase-producing (ESBL) Enterobacteriaceae, AmpC β-lactamase-producing Enterobacteriaceae (AmpC), carbapenem-resistant Enterobacteriaceae (CRE), Pseudomonas aeruginosa and Stenotrophomonas maltophilia with difficult-to-treat resistance (DTR) and carbapenem-resistant Acinetobacter baumannii (CRAB), according to the Center for Disease Control definition (https://www.cdc.gov/infectioncontrol/index.html)^14^, ^15^, ^20^. Therefore, we designed the present multicenter retrospective study, aiming at investigating, for the first time, in a wide cohort of V-V ECMOs: (i) the prevalence and incidence of MDR GN bacteria, detected by routine microbiological surveillance (i.e., rectal swabs and respiratory tract samples) or by additional biological samples collected on clinical suspicion; (ii) the risk of 1-year mortality, according to MDR GN bacteria isolation (i.e., in ‘predetected MDR GN bacteria’ group, including those patients with colonizations or infections due to MDR GN bacteria isolated before V-V ECMO cannulation; in ‘V-V ECMO-acquired’ MDR GN bacteria group; and in ‘non-MDR GN bacteria’ group, including patients never detecting MDR GN bacteria during V-V ECMO treatment); and (iii) the impact of annual hospital V-V ECMO volume on the probability of MDR GN bacteria acquisition after V-V ECMO placement.

## Methods

This multicenter observational study was conducted between January 1, 2017 and December 31, 2022 in 5 Intensive Care Units (ICU) of Italian university-affiliated hospitals, overall accounting for a total of 70-ICU beds (i.e., Mater Domini Hospital (Catanzaro); Padua University Hospital; Verona University Hospital; Policlinico University Hospital (Bari) and Fondazione IRCSS Gerardo Hospital dei Tintori Hospital (Monza)). We included adult patients, over 18 years of age, who received V-V ECMO for respiratory support during the study period. The exclusion criteria were age < 18 years old, pregnancy, veno-arterial (V-A) or mixed ECMO-configuration (e.g., V-VA), incomplete records for the main outcomes (absence of 1-year mortality and/or microbiological surveillance), and survival < 24 h after cannulation. The study was conducted in compliance with the Declaration of Helsinki and the approval for the investigation was granted by the local Ethics Committee "Comitato Etico Territoriale Regione Calabria" (approval n. 22 on September 27, 2023), which waived the need for informed consent due to the retrospective observational nature of the study. All patient data was anonymised and de-identified before analysis. This study followed the ‘Strengthening the Reporting of Observational Studies in Epidemiology (STROBE) statement guidelines for observational cohort studies’ (additional-Table 1) 21.

The decision to start V-V ECMO treatment was made by senior intensivists (PN, FL, EB, GF, LG, SG), according to the ELSO guidelines/recommendations 1. All V-V ECMOs were placed exclusively in ICU and a femoro-jugular configuration was preferred. All centers kept the ECMO circuit, as much as possible, isolated (e.g. withdrawals or infused medications were not recommended). Antimicrobial prophylaxis, at the time of cannulation, was uniformly not administered 1. Routine microbiological surveillance was uniformly conducted in all centers: rectal swabs and respiratory tract samples were collected at ICU admission and, subsequently, every 48–72 h. Blood and urine samples were collected on clinical suspicion, as well as other biological samples collected from skin, soft tissue, cannula insertion etc 15, 22. All positive microbial cultures were independently evaluated, considering the available clinical, laboratory and radiographic data, by specialized intensivists and infectious diseases specialists. The routine protocol for infection control/prevention, shared by all enrolled ICUs is reported clarified in additional-Methods 1.

To prevent the occurrence of MDR patterns, each participating center adopted an institutional antimicrobial stewardship program, which involved strict communication between ICUs and microbiology laboratories, and daily review of antibiotic regimens by dedicated infectious disease specialist consultants 23. The antimicrobial therapy was defined as ‘empiric’ when started before any microbiological evidence; or as ‘targeted’ strategy when started after microbiological evidence and according to in vitro susceptibility tests 24.

Patients were divided into three groups according to the time of MDR GN bacteria detection: (1) 'predetected MDR GN bacteria’ group, including those patients with a history of MDR GN isolation within 48 h after ECMO cannulation; (2) ‘V-V ECMO-acquired MDR GN bacteria’ group, including patients with isolation of MDR GN bacteria after 48 h from ECMO start and 48 h after disconnection 25, 26; and (3) ‘non-MDR GN bacteria’ group, including patients never culturing MDR GN bacteria during V-V ECMO support.

### Data collection

**Fig. 1:**
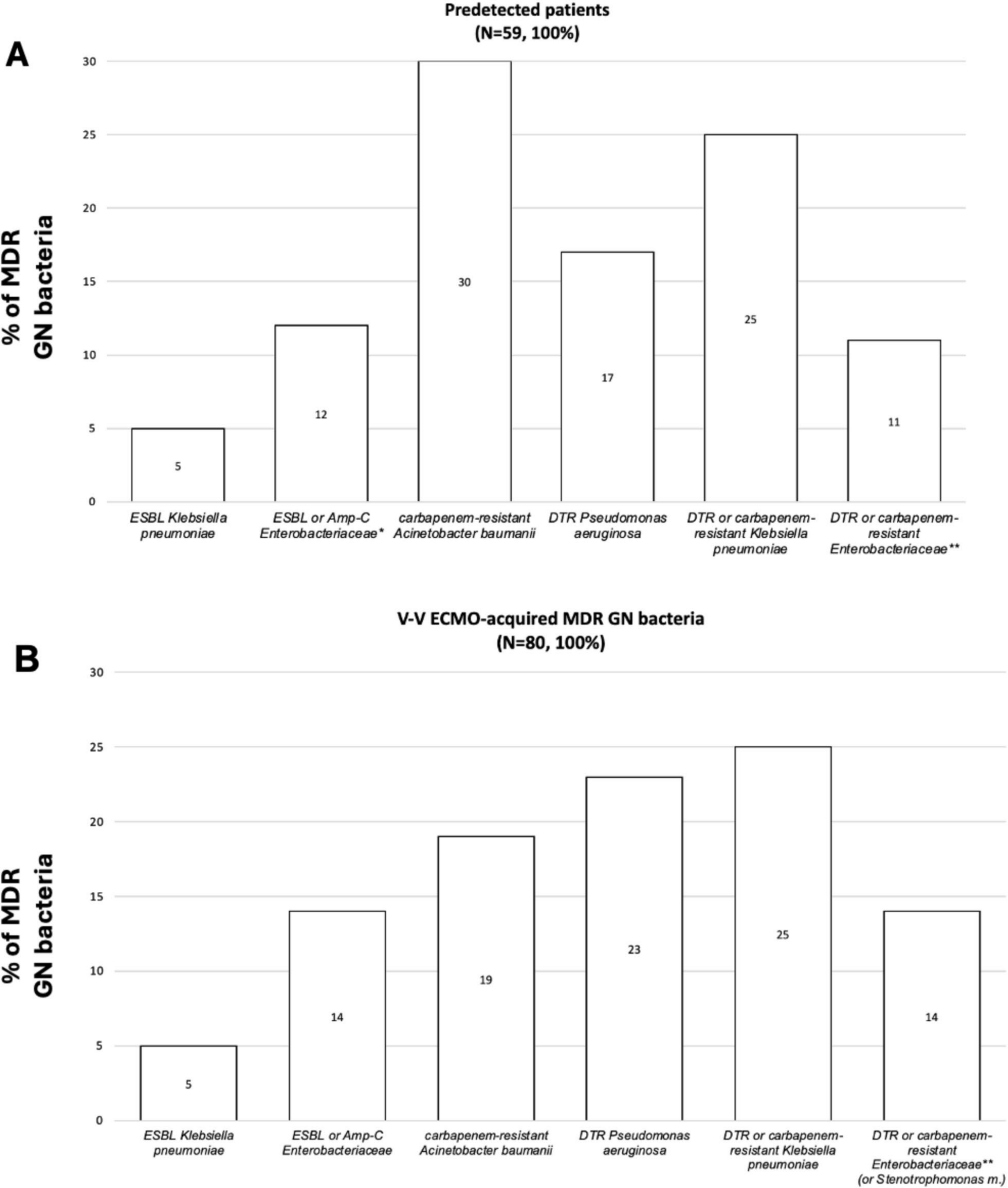
MDR GN bacteria. **A** In predetected patients. B. In ‘V-V ECMO-acquired MDR GN bacteria’ group. Data are presented as absolute frequency (% of the patients belonging to predetected MDR GN bacteria (n. 59, 100%) or as % of patients belonging to ‘V-V ECMO acquired MDR GN bacteria ‘group (n. 80, 100%). ^*^: including *Enterobacter sp., Escherichia Coli*; ^**^: including *Serratia marcescens, Enterobacter sp. and Escherichia Coli*. *Abbreviations*; MDR: multidrug resistant; GN: Gram-negative; N: number; ESBL: extended spectrum beta-lactamase; AmpC: AmpC β-lactamase-producing; DTR: difficult-to-treat resistance; sp.: species

The electronic health records were retrospectively examined and the following variables were collected: (i) demographic and baseline characteristics before V-V ECMO placement, Charlson’s Comorbidity index, Sequential Organ Failure Assessment (SOFA) score at ICU admission and at cannulation, initiation of invasive mechanical ventilation (IMV), respiratory parameters, indications for V-V ECMO, interfacility transport (defined as transfer from any medical facility outside of our ECMO centers and without ECMO capabilities 1, 27), year of V-V ECMO connection (Table 1); (ii) outcomes of interest (see full description below and in Table 2); (iii) culture results during V-V ECMO support, type of isolated bacteria, site of isolation, resistance profiles, and data on antibiotic usage (Fig. 1, Tables 3 and 4).

**Table 1:**
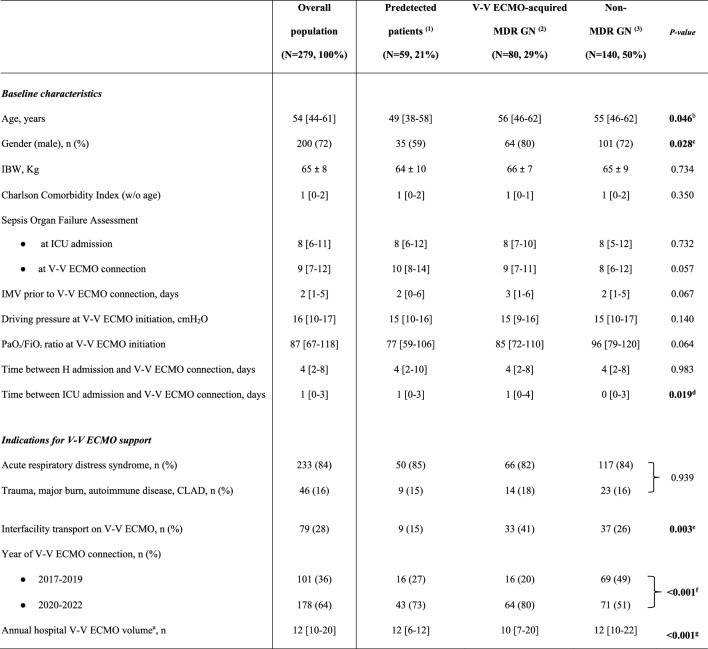
Patients’ characteristics at V-V ECMO connection

The bold font was used for significant *p*-values

Data are presented as absolute frequency (% of the included patients) or as median and [interquartile range] or as mean ± SD. 'Predetected' group includes patients, infected or colonized, by MDR GN bacteria cultured before VV-ECMO placement

^a^Annual hospital V-V ECMO volume is defined as the specific number of patients treated with V-V ECMO per year 27

^b^(1) vs (2) p-value 0.041, (1) vs (3) p-value 0.017

^c^(1) vs (2) p-value 0.013

^d^(2) vs (3) p-value 0.011

^e^(1) vs (2) p-value 0.001, (2) vs (3) p-value 0.025

^f^(1) vs (3) p-value 0.005, (2) vs (3) p-value < 0.001

^g^(1) vs (3) and (2) vs (3) p-values < 0.001

*ICU* Intensive Care Unit; *IMV* Invasive mechanical ventilation; *IBW* Ideal body weight; *ECMO* Extracorporeal membrane oxygenation; *MDR* Multidrug resistant; *GN* Gram-negative; *N or n* Number; *SD* Standard deviation; *w/o* Without; *V-V* Veno-venous; *CLAD* Chronic lung allograft dysfunction; *PaO*_*2*_*/FiO*_*2*_ The ratio of arterial oxygen partial pressure to fractional inspired oxygen

**Table 2:**
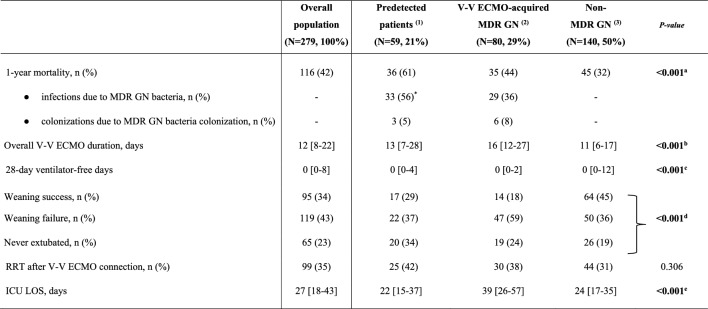
Outcomes

The bold font was used for significant *p*-values

Data are presented as absolute frequency (% of the included patients) or as median and [interquartile range]

^*^Of those non-survivors, 10 subjects were pre-infected by MDR GN bacteria at V-V ECMO initiation

^a^(1) vs (3) *p* value < 0.001

^b^(1) vs (2) *p* value < 0.001, (1) vs (3) *p* value 0.043, (2) vs (3) *p* value < 0.001

^c^(1) vs (3) *p* value 0.005, (2) vs (3) *p* value < 0.001

^d^(1) vs (2) *p* value 0.042, (1) vs (3) 0.028, (2) vs (3) *p* value < 0.001

^e^(1) vs (2) *p* value < 0.001, (2) vs (3) *p* value < 0.001

*ICU* Intensive Care Unit; *RRT* Renal replacement therapy; *IMV* Invasive mechanical ventilation; *ECMO* Extracorporeal membrane oxygenation; *MDR* Multidrug resistant; *GN* Gram-negative; *N or n* Number; *V-V* Veno-venous

**Table 3:**
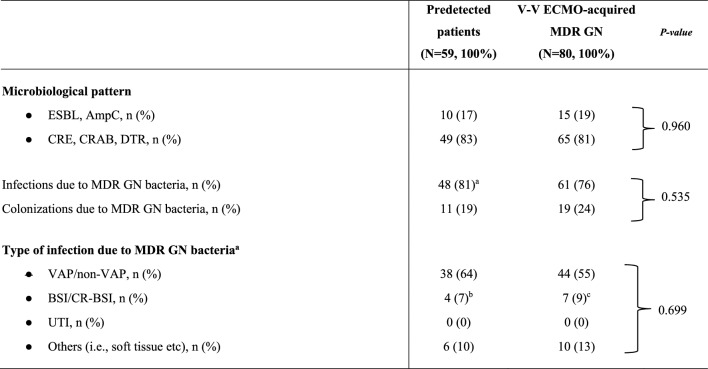
Microbiological characteristics of MDR GN bacteria

Data are presented as absolute frequency (% of the included patients)

^a^Of those patients, only 10 subjects were pre-infected by MDR GN bacteria at V-V ECMO initiation

^b^1 CR-BSI; ^c^: 2 CR-BSI. Additional information is reported in Fig. 1***.*** For more details about microbiological surveillance and diagnostic criteria see Methods and additional-Methods 1

*ECMO* extracorporeal membrane oxygenation; *MDR* Multidrug resistant; *GN* Gram-negative; *N or n* Number; *ESBL* Extended spectrum beta-lactamase; *V-V* Veno-venous; *AmpC* AmpC β-lactamase-producing; *CRE* Carbapenem-resistant Enterobacteriaceae; *DTR* Difficult-to-treat resistance (mainly Pseudomonas aeruginosa); *CRAB* Carbapenem-resistant Acinetobacter baumannii; *BSI* Blood stream infection; *VAP* Ventilator-associated pneumonia; *CR-BSI* Catheter-related bloodstream infection; *UTI* Urinary tract infection

**Table 4:**
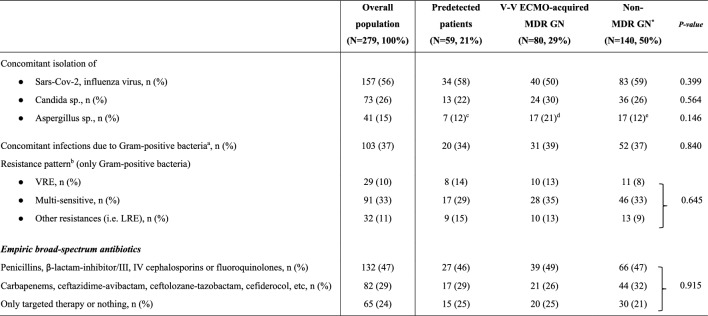
Concomitant pathogens and antibiotics

Data are presented as absolute frequency (% of the included patients) or as median and [interquartile range]. For more details about microbiological surveillance see Methods and additional-Methods 1

^*^Moreover, 39 (28%) subjects detected multisensitive GN bacteria and only 23 (16%) patients never recorded positive cultures

^a^for more details concerning Gram-positive bacteria see additional-Table 6

^b^in case of multiple bacterial isolations, only the worst resistance pattern was counted

^c^1 out of 7 patients isolated Candida sp. and Aspergillus sp. simultaneously

^d^3 out of 17 patients isolated Candida sp. and Aspergillus sp. simultaneously

^e^1 out of 7 patients isolated Candida sp. and Aspergillus sp. simultaneously

*ECMO* Extracorporeal membrane oxygenation; *MDR* Multidrug resistant; *GN* Gram-negative; *N or n* Number; *VRE* Vanco-resistant enterococcus; *LRE* Linezolid-resistant enterococcus; *V-V* Veno-venous; *sp* Species

### Outcomes

The primary outcome was assessing the rate of MDR GN bacteria in a cohort of V-V ECMOs. The MDR GN pathogens (i.e., ESBL, AmpC, CRE, DTR profiles, and CRAB), were classified according to the Center for Disease Control definition (https://www.cdc.gov/infectioncontrol/index.html)^14^ , ^15^, ^20^ and in vitro susceptibility tests (https://www.eucast.org/clinical_breakpoints). Infection was defined by organ-specific diagnostic guidelines criteria inspired by CDC/NHSN manuals (https://www.cdc.gov/nhsn/pdfs/pscmanual/17pscnosinfdef_current.pdf, see additional-Methods 1) [28–31; sepsis and septic shock was defined according to the Sepsis-3 criteria 32; while colonizations occurred in absence of clinical signs of infection 25, 26, 32. The definitions of ventilator-associated pneumonia (VAP) or non-VAP, bloodstream infection (BSI)/catheter-related bloodstream infection (CR-BSI), urinary tract infection (UTI) etc. are provided in additional-Methods 1,^15^ , ^20^, ^28^–^31^.

Other outcomes of interest included: (i) 1-year mortality; (ii) annual hospital V-V ECMO (defined as the specific number of patients treated with V-V ECMO per year 27); (iii) weaning success (defined as extubation and absence of invasive ventilatory support 48 h following extubation) and weaning failure (defined as failure of the first spontaneous breathing trial, and/or reintubation or resumption of ventilatory support within 48 h after extubation and/or death within 48 h following extubation 33); (iv) ventilation free days (VFD) (reference: 28 days) 34; (v) overall V-V ECMO duration; (vi) need of renal replacement therapy (RRT) after V-V ECMO start, and (vii) ICU length of stay (Table 2).

### Statistical analysis

Continuous variables are presented as medians and interquartile ranges [IQR] or as mean and standard deviation (SD); while categorical variables are presented as numbers (percentages). Baseline patients’ characteristics and outcome variables were compared between two or three pre-defined subpopulations, as follows: (1) ‘predetected MDR GN bacteria’ group, (2) ‘V-V ECMO-acquired MDR GN bacteria’ group, and (3) ‘non-MDR GN bacteria’ group. The sample size could not be calculated due to the explorative design of our investigation and the scarcity of data regarding the prevalence of precolonizations in patients eligible to V-V ECMO support. The t-test, Mann–Whitney test, ANOVA or Kruskal Wallis test were properly used to compare continuous variables and adjusted by Benjamini and Hochberg method. Chi-square and Fisher’s exact tests were used for comparing categorical variables.

Regarding 1-year mortality, the Kaplan Meier curves were provided only as graphical support. For investigating the risk of mortality, the unadjusted (HR) and adjusted hazard ratio (aHR), 95% confidence intervals [CI], were calculated using Cox-proportional hazards models (additional-Tables 2–4). Cox-proportional hazards models assume that the hazard ratio is constant over time, therefore the test for proportional-hazard assumption was verified for each covariate included in the univariable model. The time-dependent variable started from V-V ECMO connection for patients without MDR GN pathogens and in case of previous colonizations; while, for patients acquiring MDR GN bacteria after V-V ECMO connection, the time-dependent variable started from the first MDR GN bacteria isolation (to avoid immortal time bias). All variables described in Tables 1, 3 and 4, with a significance *p* value < 0.10, were included in an univariable Cox-proportional hazards model investigating 1-year mortality (*) (additional-Table 4). Then, as shown in additional-Tables 2 and 3***,*** the multivariable adjustment was provided according to significant confounders (*p* value < 0.05) identified through the univariable Cox-proportional hazards model, as mentioned above (*). Finally, additional analysis, exclusively focused on subjects infected by MDR GN bacteria; or only on patients with predetected MDROs, were reported on additional-Tables 2 and 4.

For investigating the impact of the annual hospital V-V ECMO volume 27 on the incidence of MDR GN bacteria acquisition after V-V ECMO connection (predetected patients were excluded from this analysis), a multivariable logistic regression was applied, after adjustment for potential confounders (*p* value < 0.05) identified through an univariable logistic regression model exclusively focused on the risk of MDR GN bacteria isolation after V-V ECMO start (additional-Table 5). The unadjusted (OR) and adjusted odds ratio (aOR), 95% CI were calculated, and all tests were two-sided and *p* values < 0.05 were considered statistically significant. The analyses were performed using R (version 4.0.3, R foundation for Statistical Computing, Vienna, Austria).

## Results

From January 2017 to December 2022, 482 ICU patients treated with V-V ECMOs for severe respiratory failure were screened. After excluding 199 subjects needing V-A or mixed ECMO-configuration, 2 patients because of incomplete records, and 2 due to a survival shorter than 24 h after V-V ECMO initiation, 279 patients (median age 54 years; 72% male) were included in the final analysis (see additional-Fig. 1). Patients’ demographic characteristics, SOFA scores, indications for V-V ECMO support and other baseline information are summarized in Table 1.

i) MDR GN bacteria detection.

In our cohort, the overall rate of MDR GN bacteria was 50%: 59 (21%) patients recorded predetected MDROs; 80 (29%) adults acquired MDR GN bacteria (generally 8 [6–11] days) after V-V ECMO cannulation; and 140 (50%) subjects had no occurrence of MDR GN bacteria during extracorporeal treatment. As described in Table 1, age, gender distribution, time of cannulation (after ICU admission) and the need for interfacility transport were differently distributed among the three subgroups of patients. Interestingly, a higher proportion of V-V ECMO (64%) has been placed after the year 2020 (*p* value < 0.001), probably due to the Sars-Cov-2 pandemic; and, similarly, the incidence of MDR GN bacteria recently increased (Table 1**)**. Forty-eight out of 59 (81%) predetected patients, and 61 out of 80 (76%) subjects belonging to the ‘V-V ECMO-acquired MDR GN bacteria’ group, developed infections due to MDR GN bacteria (Table 3). Focusing on ‘predetected MDR GN bacteria’ group, only 10 patients were pre-infected at the time of cannulation; while 38 subjected developed infections after an initial pre-colonization. According to the site of infection, VAP/non-VAP (64% vs. 55%), BSI/CR-BSI (7% vs. 9%) etc. were uniformly distributed among patients with predetected MDR GN bacteria and those subjects with V-V ECMO-acquired MDR GN bacteria, respectively (*p* value 0.0.699, see full description in Table 3). In predetected patients, the prevalent MDROs, initially isolated, were CRAB (30%) and DTR or carbapenem-resistant Klebsiella pneumoniae (25%); while, after V-V ECMO placement, the prevalent MDROs were DTR or carbapenem-resistant Klebsiella pneumoniae (25%) and DTR-Pseudomonas aeruginosa (23%), as described in Fig. 1. No differences were found considering the concomitant isolation of virus, fungi or Gram-positive (GP) bacteria (Table 4 and additional-Table 6).

ii) 1-year mortality.

**Fig. 2:**
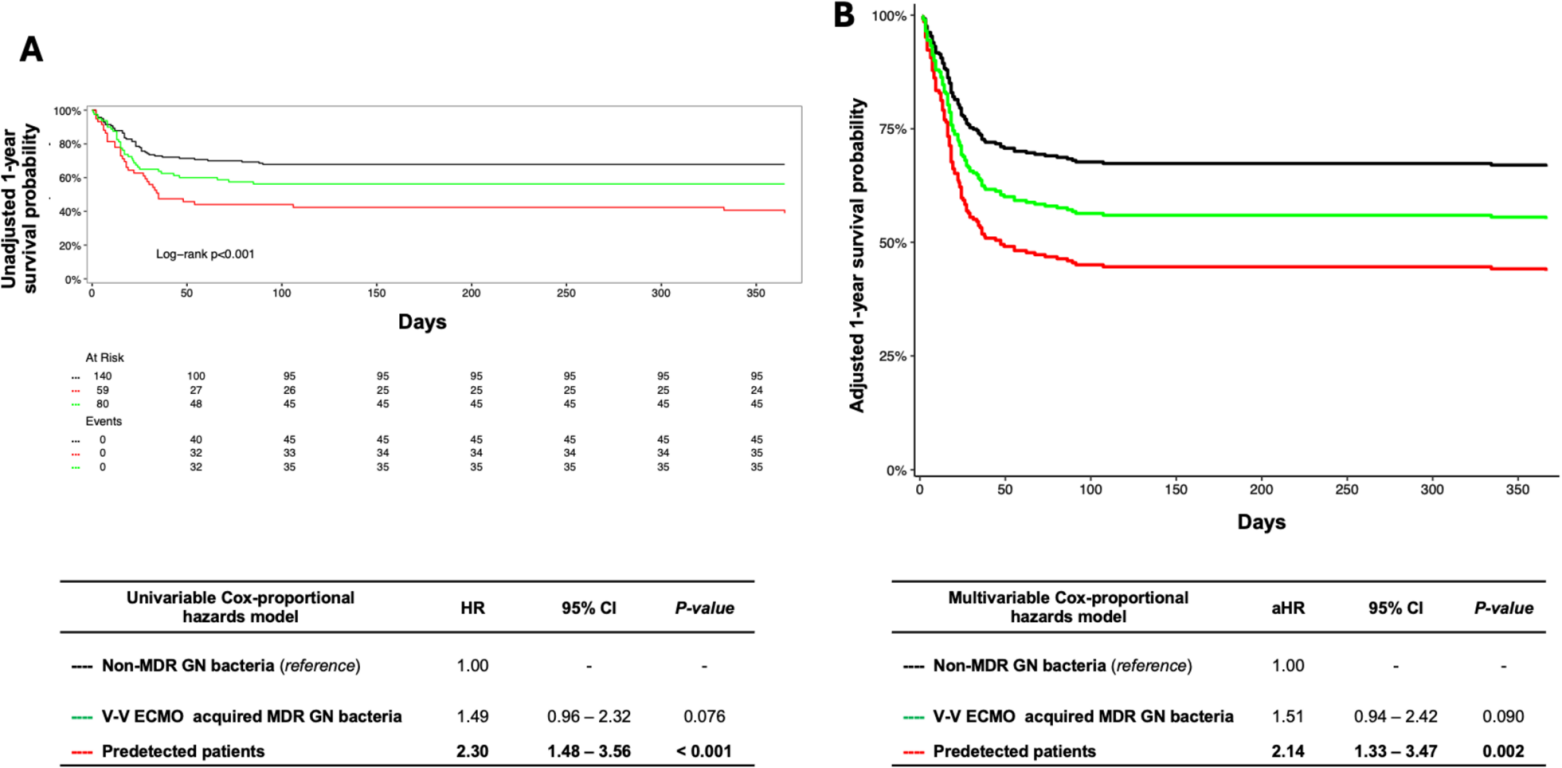
A1-year survival curves. Kaplan Meier survival curve at 1 year. The unadjusted and adjusted (covariates: age, SOFA score at V-V ECMO connection, interfacility transport, annual hospital V-V ECMO volume) HRs were calculated according to the univariable and multivariable Cox-proportional hazards models described in additional-Tables 2 and 4, respectively**.** Data are presented as HR, aHR and [95% CI]. *Abbreviations:* ECMO: extracorporeal membrane oxygenation; MDR: multidrug resistant; GN: Gram-negative; HR: hazard ratio; aHR: adjusted hazard ratio; CI: confidential interval; V-V: veno-venous

As shown in Table 2*,* the overall 1-year mortality was 42% (n. 116): 36 (61%) patients had predetected MDR GN bacteria, 35 (44%) subjects belonged to ‘V-V ECMO-acquired MDR GN bacteria’ group; and 45 (32%) adults belonged to ‘non-MDR GN bacteria’ group (*p* value < 0.001) (Fig. 2A). Indeed, predetected patients recorded a higher risk of death (aHR 2.14 [1.33–3.47], *p* value 0.002), while the ‘V-V ECMO-acquired MDR GN bacteria’ group did not (aHR 1.51 [0.94–2.42], *p* value 0.090), as compared to those patients never culturing MDR GN bacteria (*reference*) (Fig. 2B, additional-Tables 2 and 3). Similar findings were found considering only those patients experiencing infections during V-V ECMO course 25, 26, 32 (in ‘predetected’ group, aHR 2.35 [1.44–3.86] (*p* value < 0.001); in ‘V-V ECMO-acquired MDROs, aHR 1.57 [0.95–2.57] (*p* value 0.076)); or considering only pre-detected infections (n. 10) and pre-detected colonizations (n. 49) at V-V ECMO cannulation (aHR 4.44 [1.69–11.66], *p* value 0.002, and aHR 2.25 [1.02–4.98], p-value 0.044, respectively, see additional-Tables 2 and 4), as compared to those patients never culturing MDR GN bacteria (*reference*).

More information related to univariable analysis are reported in additional-Table 4.

iii) Annual hospital V-V ECMO volume.

**Fig. 3:**
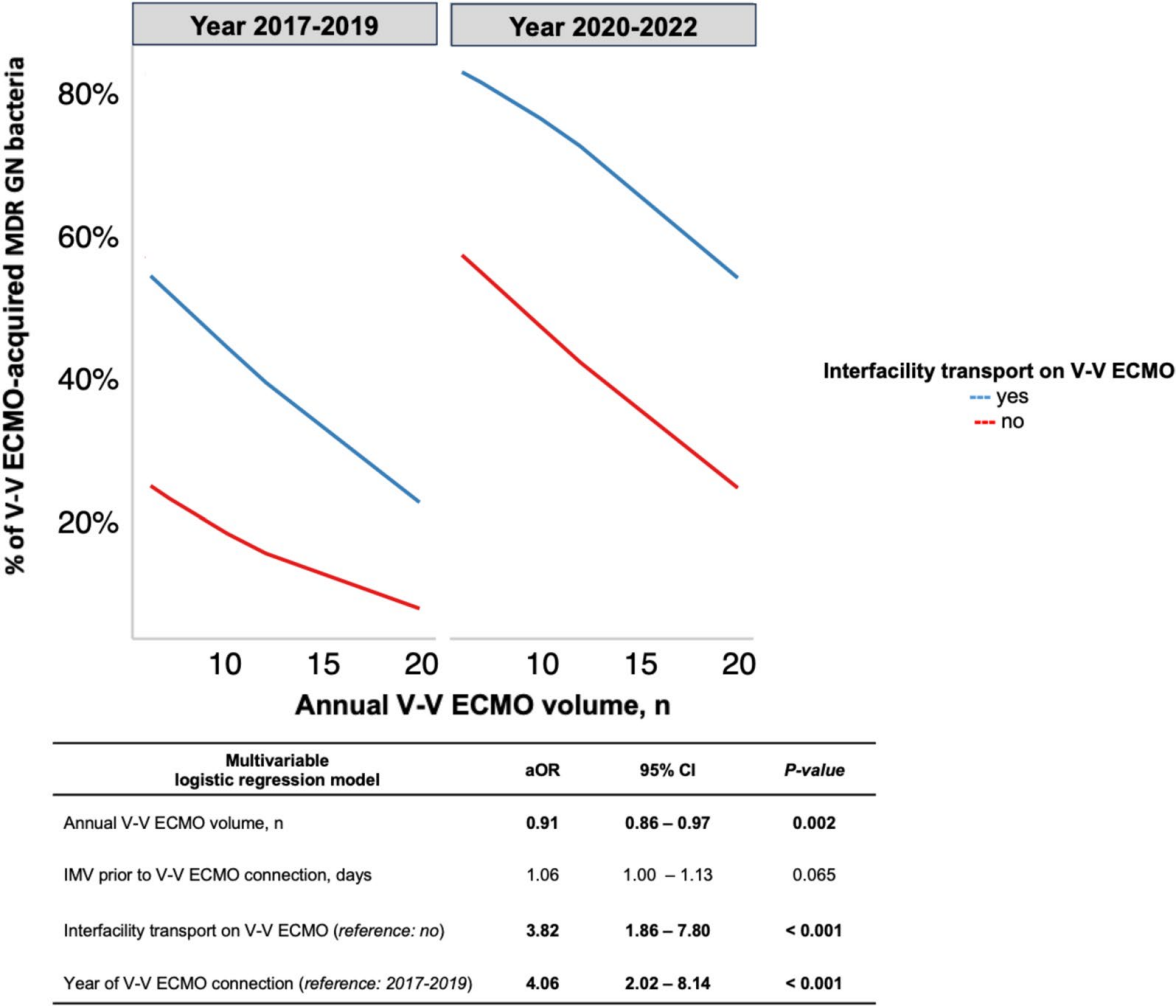
Adjusted odds of V-V ECMO-acquired MDR GN bacteria according to annual hospital V-V ECMO volume. Adjusted odds of MDR GN bacteria acquisition among patients receiving V-V ECMO support, when volume is modeled continuously. Hospital V-V ECMO volume is defined as the specific number of patients treated with V-V ECMO per year in each hospital 27. The adjusted odds of MDR GN bacteria acquisition are presented according to the results described in additional-Table 5. *Abbreviations:* ECMO: extracorporeal membrane oxygenation; MDR: multidrug resistant; GN: Gram-negative; OR: odds ratio; CI: confidential interval; IMV: invasive mechanical ventilation; V-V: veno-venous

The overall annual hospital V-V ECMO volume was 12 [10–20] per year, with a significant difference between subgroups (*p* value < 0.001) (Table 1). To note, there was a significant inverse association between the annual hospital V-V ECMO volume and the probability of acquiring MDROs after V-V ECMO connection (OR 0.91 [0.86–0.96], *p* value < 0.001) (additional-Table 5). These findings were confirmed also after adjustment for potential confounders (aOR 0.91 [0.86–0.97], *p* value 0.002), such as the need of interfacility transport and year of V-V ECMO connection, both additional risk factors for MDR GN bacteria acquisition (Fig. 3).

iv) Secondary outcomes.

The overall duration of V-V ECMO and ICU LOS were longer in ‘V-V ECMO-acquired MDR GN bacteria’ group, as compared to the other subgroups (both *p* values < 0.001); while the successful liberation from IMV was more frequent, and 28-day VFD were longer, in those patients never experiencing MDR GN bacteria (both *p* values < 0.001) (Table 2). A full description of all outcomes of interest is reported in Table 2.

## Discussion

In this multicenter retrospective study, we found that among 279 consecutive adult patients supported by V-V ECMO for acute respiratory failure, the prevalence of MDR GN colonization at V-V ECMO placement was 21%, while the incidence was 29% among patients acquiring MDR GN bacteria during their V-V ECMO course. In the overall population, 1-year mortality was 42%, with a higher risk of death in patients with MDR GN detection before V-V ECMO cannulation (61%) compared to those patients with V-V ECMO-acquired MDR GN bacteria (44%) or with absent MDR GN bacteria (32%). Same findings were found considering only infections. Indeed, to the best of our knowledge, pre-existing MDR GN bacteria isolations, have never been demonstrated as an independent risk factor correlated with poor survival in patients requiring V-V ECMO, highlighting a clear association, rather than a causality, between MDR GN bacteria predetection and mortality. Furthermore, we showed that the risk of acquiring MDR GN bacteria, during ECMO treatment, was greater in centers with lower annual hospital V-V ECMO volume, also after adjustment for potential confounders such as the need of interfacility transport and year of V-V ECMO placement.

In the last decade, a worrying burden of GN bacteria with high levels of antimicrobial resistance has been reported, especially in the critical care setting where MDR GN bacteria are isolated in a high percentage of patients 24, 35–38. Confirming this alarming trend, our study showed that MDR GN bacteria were isolated in almost half of the patients, either before or after V-V ECMO connection, with a clear increase in the last years. To date, based on the most recent literature, the incidence of MDR GN pathogens during V-V ECMO support is still unclear and no epidemiological data are available on this specific population for providing a reliable comparison with our study. According to the ELSO registry, that includes data of both V-A and V-V ECMO from all ELSO centers worldwide, GN pathogens, in particular Pseudomonas aeruginosa and Enterobacteriaceae, are among the most common bacteria isolated during ECMO support, second only to coagulase-negative Staphylococci (more prevalent in V-A ECMO) 37. In line with our findings, Grasselli et al. reported, in a cohort of 90 non-surgical patients undergoing V-A and V-V ECMO, an overall incidence of GN bacteria of 48% after ECMO connection 17. Of those, 60% of GN bacteria were classified as MDROs, while no data were reported about pre-existing isolations of MDROs 17. An even higher incidence was described by Gao et al., who retrospectively investigated a Chinese cohort of 109 patients receiving ECMO from 2014 to 2022: in the subgroup of 29 patients supported by V-V ECMO, the incidence of MDR-GN bacteria was 78% after ECMO placement 39. In line with our study, where MDR GN bacteria were collected mainly from airway samples, several previous single-center experiences defined GN bacteria, especially Enterobacteriaceae, as the major determinant of respiratory infections in patients requiring V-V ECMO 40, 41, being MDR GN pathogens the cause of the 35% of cases of VAP during ECMO support 17. These epidemiological results should not be surprising, since ECMO patients present several risk factors for the development of infectious complications. In fact, the extreme severity of illness seems to be associated with the risk of difficult-to-treat bacteria detection 42. Both invasiveness of care, with several paracorporeal devices (e.g., tracheal intubation, vascular lines, urine catheter, drainages), and critical illness itself contribute to alter the patients’ microbiota and to increase susceptibility to bacterial isolations [43–45. In particular, a diagnosis of MDR GN bacteria, prior to V-V ECMO placement, seems to be a marker of pre-existing overall illness burden rather than a discrete disease entity causing mortality. To note, not only pre-infected patients, but also pre-colonized subjects recorded a higher risk of death, probably justified by a great frailty of predetected patients and a high susceptibility to progress from colonization to overt MDR GN bacteria-related infections. So, despite the need for further well-designed studies for confirming the negative impact of pre-existing MDROs on patients’ survival, we believe that all patients, eligible to V-V ECMO treatment, should be microbiologically screened (i.e. rectal swabs), both for better defining the risk of death, and for encouraging a strict clinical monitoring and follow-up, especially among predetected MDR GN patients, with the aim to avoid any progress from colonization to overt infections.

Furthermore, ECMO patients are frequently exposed to broad-spectrum antibiotics, which impose a selection pressure favoring the emergence of antimicrobial resistances 9, 38, 46. However, in keeping with the findings of Grasselli et al. 17, 38, in our cohort the empiric exposure to carbapenems or to other broad-spectrum antibiotics was remarkably low and equally distributed among subpopulations. This result may be ascribable both to the encouragement of adopting carbapenem-sparing targeted strategies rather than empiric broad-spectrum therapies 47, 48, and to the implementation of an ICU-dedicated antimicrobial stewardship program in all centers participating in our study, aiming at promoting the prompt selection of optimal (hopefully targeted) antimicrobial regimens 23. Therefore, our results do not support the use of broad-spectrum antimicrobial prophylaxis during V-V ECMO placement, in line with the last ELSO guidelines 1, but may suggest a proactive behavior, with an early identification of infections and a prompt administration of targeted therapies, for limiting the development of difficult-to-treat resistances.

Interestingly, in heterogeneous populations, including either adult or pediatric patients and both V-A and V-V ECMO, infectious complications during extracorporeal support were associated with an increased risk of death, ranging from 38 to 63% 37, 38, 49. To note, our study, exclusively focused on V-V ECMOs, newly recorded the highest risk of death among predetected subjects. This finding may underline the great importance of applying standardized precautions for preventing the development of ‘difficult-to-treat’ infectious complications and the need for most current eligibility criteria for V-V ECMO in light of a significant increase of MDROs in a few years. Moreover, our findings showed that many patients culturing MDR GN bacteria during V-V ECMO support (and not exclusively before) recorded poorer secondary outcomes, as compared to those subjects never detecting MDROs 38, 41, 50, 51. Probably, these results reflect the need for a higher invasiveness of treatment in these specific subgroups of patients, likely due to a higher degree of critical illness, as already shown in previous investigations 17, 41.

Finally, inspired by Barbaro et al., who reported, in 10.588 adult patients receiving ECMO, a significantly higher risk of mortality for those patients who were treated at hospitals with annual-volume < 6 ECMO cases for year, we hypothesized (and showed) that a low annual hospital V-V ECMO volume increased the probability of V-V ECMO-acquired MDR GN bacteria 27. These findings highlight the strong relationship existing between effective microbiological surveillance programs and a high ICU-quality, usually provided by expert ECMO-teams/centers 52.

This study had several limitations. First, it is a retrospective observational study which bears the limits of this design. Second, despite a wide population consisting exclusively of V-V ECMO adult patients, the categorization of the cohort according to the MDR GN isolation status inevitably resulted in three small-size subgroups. We believe that a broader cohort in the future would better delineate outcomes and validate our findings. Third, we marginally described the impact of virus, fungi and GP bacteria on our cohort, first, because out of our primary aim and, second, because vancomycin-resistant Enterococci are more prevalent during V-A ECMO 11, 12, 37 and have been recently described in declining in ICU patients 53, 54. However, several information concerning virus, fungi and GP pathogens are reported in Table 4 and additional-Table 6. Fourth, we did not investigate whether the cannulation site may influence the infectious risk, despite the internal jugular and femoral vein being the most common sites of catheterization (> 80% in our cohort) 55. Fifth, according to our findings, describing an inverse relationship between MDR GN bacteria occurrence and local institutional experience, we cannot exclude also a higher rate of other complications, in addition to infectious ones, occurring in those centers with a lower annual hospital V-V ECMO volume. Sixth, although we believe that the comparison of MDR acquisition between ECMO and non-ECMO patients would be extremely interesting, we believe that such an analysis is far beyond the aim of the present study and would deserve a dedicated study protocol.

## Conclusions

In conclusion, in this multicenter retrospective study investigating the prevalence and incidence of MDR GN bacteria in V-V ECMO adult patients, the isolation of MDR GN bacteria was 21% before and 29% after V-V ECMO connection (overall rate: 50%). We reported an overall 1-year mortality of 42%, with a higher risk of mortality in predetected patients. Finally, a larger annual hospital V-V ECMO volume was associated with a lower probability of acquiring MDR GN bacteria during ECMO treatment.

## Home point

**Study question:** Could a pre-existing isolation of MDR GN bacteria, or the acquisition during V-V ECMO support, affect patient’s survival at 1 year? What about the annual hospital V-V ECMO volume on the risk of acquiring MDR GN pathogens?

**Results:** 1-year mortality is higher in patients with pre-existing history of MDR GN bacteria, while not in those patients acquiring MDR GN bacteria after V-V ECMO placement. Similar findings were found considering only infections. A larger annual hospital V-V ECMO volume is associated with a lower probability of acquiring MDR GN bacteria.

**Interpretation:** 21% of MDR GN bacteria was detected before and 29% after V-V ECMO connection. A previous history of MDR GN bacteria prior to V-V ECMO was an independent risk factor for mortality, also when only infections were considered. The annual hospital V-V ECMO volume affected the probability of acquiring MDR GN bacteria.

## Supplementary Information

Additional file1

## Data Availability

No datasets were generated or analysed during the current study.

## Abbreviations

ARDS: Acute respiratory distress syndrome
aOR: Adjusted odds ratio
AmpC: AmpC β-lactamase-producing
BMI: Body mass index
BSI: Bloodstream infection
CR-BSI: Catheter-related bloodstream infection
CRAB: Carbapenem-resistant *Acinetobacter baumannii*
CREL: Carbapenem-resistant *Enterobacteriaceae*
CLADL: Chronic lung allograft dysfunction
CIL: Confidence interval
DTR: Difficult-to-treat resistance
ESBL: Extended-spectrum β-lactamase-producing
ESBL: Extended-spectrum beta-lactamase
ECMO: Extracorporeal membrane oxygenation
GN: Gram-negative
GP: Gram-positive
HR: Hazard ratio
IBW: Ideal body weight
IR: Incidence rate
FiO2: Inspiratory fraction of oxygen
ICU: Intensive care unit
IMV: Invasive mechanical ventilation
MDR: Multi-drug resistant
MDRO: Multi-drug resistant organism
N or n: Number
OR: Odds ratio
RRT: Renal replacement therapy
SOFA: Sequential Organ Failure Assessment
sp.: Species
SD: Standard deviation
UTI: Urinary tract infection
V-V ECMO: Veno-venous extracorporeal membrane oxygenation
VAP: Ventilator-associated pneumonia
w/o: Without

## References

[1] Tonna JE, Abrams D, Brodie D, Greenwood JC, Rubio Mateo-Sidron JA, Usman A, et al. Management of adult patients supported with venovenous extracorporeal membrane oxygenation (VV ECMO): guideline from the extracorporeal life support organization (ELSO). ASAIO J Am Soc Artif Intern Organs. 2021;67(6):601–10. 10.1097/MAT.0000000000001432 PMC8315725 33965970

[2] Bellani G, Laffey JG, Pham T, Fan E, Brochard L, Esteban A, et al. Epidemiology, patterns of care, and mortality for patients with acute respiratory distress syndrome in intensive care units in 50 countries. JAMA. 2016;315(8):788–800. 26903337 10.1001/jama.2016.0291

[3] Grasselli G, Calfee CS, Camporota L, Poole D, Amato MBP, Antonelli M, et al. ESICM guidelines on acute respiratory distress syndrome: definition, phenotyping and respiratory support strategies. Intensive Care Med Intensive Care Med. 2023;49(7):727–59. 37326646 10.1007/s00134-023-07050-7 PMC10354163

[4] Definition Task Force ARDS, Ranieri VM, Rubenfeld GD, Thompson BT, Ferguson ND, Caldwell E, et al. Acute respiratory distress syndrome: the Berlin Definition. JAMA. 2012;307(23):2526–33. 22797452 10.1001/jama.2012.5669

[5] Tonna JE, Boonstra PS, MacLaren G, Paden M, Brodie D, Anders M, et al. Extracorporeal life support organization registry international report 2022: 100,000 Survivors. ASAIO J. 2024;70(2):131–43. 38181413 10.1097/MAT.0000000000002128 PMC10962646

[6] Peek GJ, Mugford M, Tiruvoipati R, Wilson A, Allen E, Thalanany MM, et al. Efficacy and economic assessment of conventional ventilatory support versus extracorporeal membrane oxygenation for severe adult respiratory failure (CESAR): a multicentre randomised controlled trial. Lancet Lond Engl. 2009;374(9698):1351–63. 10.1016/S0140-6736(09)61069-2 19762075

[7] Sella N, Pettenuzzo T, Zarantonello F, Andreatta G, De Cassai A, Schiavolin C, et al. Electrical impedance tomography: a compass for the safe route to optimal PEEP. Respir Med. 2021;187: 106555. 34352563 10.1016/j.rmed.2021.106555

[8] Brodie D, Bacchetta M. Extracorporeal membrane oxygenation for ARDS in adults. N Engl J Med. 2011;365(20):1905–14. 22087681 10.1056/NEJMct1103720

[9] Biffi S, Di Bella S, Scaravilli V, Peri AM, Grasselli G, Alagna L, et al. Infections during extracorporeal membrane oxygenation: epidemiology, risk factors, pathogenesis and prevention. Int J Antimicrob Agents. 2017;50(1):9–16. 28528989 10.1016/j.ijantimicag.2017.02.025

[10] Schmidt M, Bailey M, Sheldrake J, Hodgson C, Aubron C, Rycus PT, et al. Predicting survival after extracorporeal membrane oxygenation for severe acute respiratory failure. The respiratory extracorporeal membrane oxygenation survival prediction (RESP) score. Am J Respir Crit Care Med. 2014;189(11):1374–82. 24693864 10.1164/rccm.201311-2023OC

[11] Menaker J, Galvagno S, Rabinowitz R, Penchev V, Hollis A, Kon Z, et al. Epidemiology of blood stream infection in adult extracorporeal membrane oxygenation patients: a cohort study. Heart Lung. 2019;48(3):236–9. 30686618 10.1016/j.hrtlng.2019.01.004

[12] Yang L, Li M, Gu S, Feng Y, Huang X, Zhang Y, et al. Risk factors for bloodstream infection (BSI) in patients with severe acute respiratory distress syndrome (ARDS) supported by veno-venous extracorporeal membrane oxygenation (VV-ECMO). BMC Pulm Med. 2022;22(1):370. 36171599 10.1186/s12890-022-02164-y PMC9518943

[13] Paul M, Carrara E, Retamar P, Tängdén T, Bitterman R, Bonomo RA, et al. European society of clinical microbiology and infectious diseases (ESCMID) guidelines for the treatment of infections caused by multidrug-resistant Gram-negative bacilli (endorsed by European society of intensive care medicine). Clin Microbiol Infect Off Publ Eur Soc Clin Microbiol Infect Dis. 2022;28(4):521–47. 10.1016/j.cmi.2021.11.025 34923128

[14] Rossolini GM, Bochenska M, Fumagalli L, Dowzicky M. Trends of major antimicrobial resistance phenotypes in enterobacterales and gram-negative non-fermenters from ATLAS and EARS-net surveillance systems: Italian vs. European and global data, 2008–2018. Diagn Microbiol Infect Dis. 2021;101(4): 115512. 34419741 10.1016/j.diagmicrobio.2021.115512

[15] Tiseo G, Brigante G, Giacobbe DR, Maraolo AE, Gona F, Falcone M, et al. Diagnosis and management of infections caused by multidrug-resistant bacteria: guideline endorsed by the Italian Society of Infection and Tropical Diseases (SIMIT), the Italian Society of Anti-Infective Therapy (SITA), the Italian Group for Antimicrobial Stewardship (GISA), the Italian Association of Clinical Microbiologists (AMCLI) and the Italian Society of Microbiology (SIM). Int J Antimicrob Agents. 2022;60(2): 106611. 35697179 10.1016/j.ijantimicag.2022.106611

[16] Grasselli G, Scaravilli V, Mangioni D, Scudeller L, Alagna L, Bartoletti M, et al. Hospital-acquired infections in critically ill patients with COVID-19. Chest. 2021;160(2):454–65. 33857475 10.1016/j.chest.2021.04.002 PMC8056844

[17] Grasselli G, Scaravilli V, Di Bella S, Biffi S, Bombino M, Patroniti N, et al. Nosocomial infections during extracorporeal membrane oxygenation: incidence, etiology, and impact on patients’ outcome. Crit Care Med. 2017;45(10):1726–33. 28777198 10.1097/CCM.0000000000002652

[18] Azim A, Dwivedi M, Rao PB, Baronia AK, Singh RK, Prasad KN, et al. Epidemiology of bacterial colonization at intensive care unit admission with emphasis on extended-spectrum beta-lactamase- and metallo-beta-lactamase-producing Gram-negative bacteria–an Indian experience. J Med Microbiol. 2010;59(Pt 8):955–60. 20413621 10.1099/jmm.0.018085-0

[19] Barbier F, Bailly S, Schwebel C, Papazian L, Azoulay É, Kallel H, et al. Infection-related ventilator-associated complications in ICU patients colonised with extended-spectrum β-lactamase-producing Enterobacteriaceae. Intensive Care Med. 2018;44(5):616–26. 29663045 10.1007/s00134-018-5154-4

[20] Tamma PD, Aitken SL, Bonomo RA, Mathers AJ, van Duin D, Clancy CJ. Infectious diseases society of America 2023 guidance on the treatment of antimicrobial resistant gram-negative infections. Clin Infect Dis. 2023. 10.1093/cid/ciad428. 37463564 10.1093/cid/ciad428

[21] von Elm E, Altman DG, Egger M, Pocock SJ, Gøtzsche PC, Vandenbroucke JP, et al. The strengthening the reporting of observational studies in epidemiology (STROBE) statement: guidelines for reporting observational studies. PLoS Med. 2007;4(10): e296. 17941714 10.1371/journal.pmed.0040296 PMC2020495

[22] Klompas M, Branson R, Cawcutt K, Crist M, Eichenwald EC, Greene LR, et al. Strategies to prevent ventilator-associated pneumonia, ventilator-associated events, and nonventilator hospital-acquired pneumonia in acute-care hospitals: 2022 Update. Infect Control Hosp Epidemiol. 2022;43(6):687–713. 35589091 10.1017/ice.2022.88 PMC10903147

[23] Pickens CI, Wunderink RG. Principles and practice of antibiotic stewardship in the ICU. Chest. 2019;156(1):163–71. 30689983 10.1016/j.chest.2019.01.013 PMC7118241

[24] Boscolo A, Sella N, Pettenuzzo T, De Cassai A, Crociani S, Schiavolin C, et al. Multidrug-resistant and extended-spectrum β-lactamase gram-negative bacteria in bilateral lung transplant recipients: incidence, risk factors, and in-hospital mortality. Chest. 2022;162(6):1255–64. 35850288 10.1016/j.chest.2022.06.046

[25] Paling FP, Wolkewitz M, Bode LGM, Klein Klouwenberg PMC, Ong DSY, Depuydt P, de Bus L, Sifakis F, Bonten MJM, Kluytmans JAJW. Staphylococcus aureus colonization at ICU admission as a risk factor for developing S. aureus ICU pneumonia. Clin Microbiol Infect. 2017;23(1):49.e9-49.e14. 27693658 10.1016/j.cmi.2016.09.022

[26] Paling FP, Hazard D, Bonten MJM, Goossens H, Jafri HS, Malhotra-Kumar S, Sifakis F, Weber S, Kluytmans JAJW. Association of staphylococcus aureus colonization and pneumonia in the intensive care unit. JAMA Netw Open. 2020;3(9):e2012741. 32997125 10.1001/jamanetworkopen.2020.12741 PMC7527877

[27] Barbaro RP, Odetola FO, Kidwell KM, Paden ML, Bartlett RH, Davis MM, et al. Association of hospital-level volume of extracorporeal membrane oxygenation cases and mortality. Analysis of the extracorporeal life support organization registry. Am J Respir Crit Care Med. 2015;191(8):894–901. 25695688 10.1164/rccm.201409-1634OC PMC4435456

[28] Horan TC, Andrus M, Dudeck MA. CDC/NHSN surveillance definition of health care-associated infection and criteria for specific types of infections in the acute care setting. Am J Infect Control. 2008;36(5):309–32. 18538699 10.1016/j.ajic.2008.03.002

[29] Mermel LA, Allon M, Bouza E, Craven DE, Flynn P, O’Grady NP, et al. Clinical practice guidelines for the diagnosis and management of intravascular catheter-related infection: 2009 Update by the Infectious Diseases Society of America. Clin Infect Dis. 2009;49(1):1–45. 19489710 10.1086/599376 PMC4039170

[30] Hooton TM, Bradley SF, Cardenas DD, Colgan R, Geerlings SE, Rice JC, et al. Diagnosis, prevention, and treatment of catheter-associated urinary tract infection in adults: 2009 international clinical practice guidelines from the infectious diseases society of America. Clin Infect Dis. 2010;50(5):625–63. 20175247 10.1086/650482

[31] American Thoracic Society; Infectious Diseases Society of America. Guidelines for the management of adults with hospital-acquired, ventilator-associated, and healthcare-associated pneumonia. Am J Respir Crit Care Med. 2005;171(4):388–416. 15699079 10.1164/rccm.200405-644ST

[32] Singer M, Deutschman CS, Seymour CW, Shankar-Hari M, Annane D, Bauer M, et al. The third international consensus definitions for sepsis and septic shock (Sepsis-3). JAMA. 2016;315(8):801–10. 26903338 10.1001/jama.2016.0287 PMC4968574

[33] Heunks LM, van der Hoeven JG. Clinical review: the ABC of weaning failure–a structured approach. Crit Care Lond Engl. 2010;14(6):245. 10.1186/cc9296 PMC3220047 21143773

[34] Renard Trichè L, Futier E, De Carvalho M, Pinol-Domenech N, Bodet-Contentin L, Jabaudon M, et al. Sample size estimation in clinical trials using ventilator-free days as the primary outcome: a systematic review. Crit Care. 2023;27(1):303. 37528425 10.1186/s13054-023-04562-y PMC10394791

[35] Vincent JL, Rello J, Marshall J, Silva E, Anzueto A, Martin CD, et al. International study of the prevalence and outcomes of infection in intensive care units. JAMA. 2009;302(21):2323–9. 19952319 10.1001/jama.2009.1754

[36] Gupta R, Malik A, Rizvi M, Ahmed M, Singh A. Epidemiology of multidrug-resistant Gram-negative pathogens isolated from ventilator-associated pneumonia in ICU patients. J Glob Antimicrob Resist. 2017;9:47–50. 28288860 10.1016/j.jgar.2016.12.016

[37] Bizzarro MJ, Conrad SA, Kaufman DA, Rycus P. Extracorporeal life support organization task force on infections, extracorporeal membrane oxygenation. Infections acquired during extracorporeal membrane oxygenation in neonates, children, and adults. Pediatr Crit Care Med J Soc Crit Care Med World Fed Pediatr Intensive Crit Care Soc. 2011;12(3):277–81. 10.1097/PCC.0b013e3181e28894 20495508

[38] Grasselli G, Scaravilli V, Alagna L, Bombino M, De Falco S, Bandera A, et al. Gastrointestinal colonization with multidrug-resistant Gram-negative bacteria during extracorporeal membrane oxygenation: effect on the risk of subsequent infections and impact on patient outcome. Ann Intensive Care. 2019;9(1):141. 31853672 10.1186/s13613-019-0615-7 PMC6920277

[39] Gao X, Wang W. The etiological and drug resistance characteristics of multidrug-resistant pathogens in patients requiring extracorporeal membrane oxygenation: a retrospective cohort study. Infect Drug Resist. 2023;16:4929–41. 37546369 10.2147/IDR.S421413 PMC10402724

[40] Aubron C, Cheng AC, Pilcher D, Leong T, Magrin G, Cooper DJ, et al. Infections acquired by adults who receive extracorporeal membrane oxygenation: risk factors and outcome. Infect Control Hosp Epidemiol. 2013;34(1):24–30. 23221189 10.1086/668439

[41] Schmidt M, Bréchot N, Hariri S, Guiguet M, Luyt CE, Makri R, et al. Nosocomial infections in adult cardiogenic shock patients supported by venoarterial extracorporeal membrane oxygenation. Clin Infect Dis Off Publ Infect Dis Soc Am. 2012;55(12):1633–41. 10.1093/cid/cis783 PMC3888098 22990851

[42] Gasink LB, Edelstein PH, Lautenbach E, Synnestvedt M, Fishman NO. Risk factors and clinical impact of Klebsiella pneumoniae carbapenemase-producing K. pneumoniae. Infect Control Hosp Epidemiol. 2009;30(12):1180–5. 19860564 10.1086/648451 PMC2893218

[43] Zhou HY, Yuan Z, Du YP. Prior use of four invasive procedures increases the risk of Acinetobacter baumannii nosocomial bacteremia among patients in intensive care units: a systematic review and meta-analysis. Int J Infect Dis IJID Off Publ Int Soc Infect Dis. 2014;22:25–30. 10.1016/j.ijid.2014.01.018 24607429

[44] Lankelma JM, van Vught LA, Belzer C, Schultz MJ, van der Poll T, de Vos WM, et al. Critically ill patients demonstrate large interpersonal variation in intestinal microbiota dysregulation: a pilot study. Intensive Care Med. 2017;43(1):59–68. 27837233 10.1007/s00134-016-4613-z PMC5203863

[45] Wolff NS, Hugenholtz F, Wiersinga WJ. The emerging role of the microbiota in the ICU. Crit Care Lond Engl. 2018;22(1):78. 10.1186/s13054-018-1999-8 PMC5861706 29559006

[46] Kao LS, Fleming GM, Escamilla RJ, Lew DF, Lally KP. Antimicrobial prophylaxis and infection surveillance in extracorporeal membrane oxygenation patients: a multi-institutional survey of practice patterns. ASAIO J. 2011;57(3):231–8. 21317768 10.1097/MAT.0b013e31820d19ab

[47] Yusuf E, Bax HI, Verkaik NJ, van Westreenen M. An update on eight ‘new’ antibiotics against multidrug-resistant gram-negative bacteria. J Clin Med. 2021;10(5):1068. 33806604 10.3390/jcm10051068 PMC7962006

[48] Sherwin J, Heath T, Watt K. Pharmacokinetics and dosing of anti-infective drugs in patients on extracorporeal membrane oxygenation: a review of the current literature. Clin Ther. 2016;38(9):1976–94. 27553752 10.1016/j.clinthera.2016.07.169 PMC5535730

[49] Vogel AM, Lew DF, Kao LS, Lally KP. Defining risk for infectious complications on extracorporeal life support. J Pediatr Surg. 2011;46(12):2260–4. 22152861 10.1016/j.jpedsurg.2011.09.013

[50] Sun HY, Ko WJ, Tsai PR, Sun CC, Chang YY, Lee CW, et al. Infections occurring during extracorporeal membrane oxygenation use in adult patients. J Thorac Cardiovasc Surg. 2010;140(5):1125-1132.e2. 20708754 10.1016/j.jtcvs.2010.07.017

[51] Pieri M, Agracheva N, Fumagalli L, Greco T, De Bonis M, Calabrese MC, et al. Infections occurring in adult patients receiving mechanical circulatory support: the two-year experience of an Italian National Referral Tertiary Care Center. Med Intensiva. 2013;37(7):468–75. 23040766 10.1016/j.medin.2012.08.009

[52] Cheng W, Chen J, Ma X, Sun J, Gao S, Wang Y, et al. Association between ICU quality and in-hospital mortality of V-V ECMO-supported patients-the ECMO quality improvement action (EQIA) study: a national cohort study in China from 2017 to 2019. Front Med. 2023;18(2):315–26. 37991709 10.1007/s11684-023-1014-x

[53] Lemmen SW, Häfner H, Zolldann D, Stanzel S, Lütticken R. Distribution of multi-resistant gram-negative versus gram-positive bacteria in the hospital inanimate environment. J Hosp Infect. 2004;56(3):191–7. 15003666 10.1016/j.jhin.2003.12.004

[54] Vincent JL, Sakr Y, Singer M, Martin-Loeches I, Machado FR, Marshall JC, et al. Prevalence and outcomes of infection among patients in intensive care units in 2017. JAMA. 2020;323(15):1478–87. 32207816 10.1001/jama.2020.2717 PMC7093816

[55] Crivellari M, Pappalardo F. Femoro-jugular cannulation in veno-venous extracorporeal membrane oxygenation PRO/CON. J Thorac Dis. 2018;10(Suppl 5):S613–5. 29732178 10.21037/jtd.2018.02.89 PMC5911552

