# Correction to: Retrospective ANalysis of multi-drug resistant Gram-nEgative bacteRia on veno-venous extracorporeal membrane oxygenation. The multicenter RANGER STUDY

**DOI:** 10.1186/s13054-024-05231-4

**Published:** 2025-02-13

**Authors:** Annalisa Boscolo, Andrea Bruni, Marco Giani, Eugenio Garofalo, Nicolò Sella, Tommaso Pettenuzzo, Michela Bombino, Matteo Palcani, Emanuele Rezoagli, Matteo Pozzi, Elena Falcioni, Elisa Pistollato, Eugenio Biamonte, Francesco Murgolo, Graziella D’Arrigo, Mercedes Gori, Giovanni Luigi Tripepi, Leonardo Gottin, Federico Longhini, Salvatore Grasso, Paolo Navalesi, Giuseppe Foti

**Affiliations:** 1 Department of Medicine (DIMED), University of Padua, 13 Gallucci Street, 35121 Padua, Italy; 2 Institute of Anesthesia and Critical Care, Padua University Hospital, Padua, Italy; 3 Department of Cardiac, Thoracic, Vascular Sciences and Public Health, University of Padua, Padua, Italy; 4 Department of Medical and Surgical Sciences, Magna Graecia University, Catanzaro, Italy; 5 School of Medicine and Surgery, University of Milano-Bicocca, Monza, Italy; 6 Department of Emergency and Critical Care, IRCSS San Gerardo Dei Tintori, Monza, Italy; 7 Department of Surgery, Dentistry, Paediatrics and Gynaecology, University of Verona, Verona, Italy; 8 Cardiothoracic and Vascular Intensive Care Unit, Verona University Hospital, Verona, Italy; 9 Department of Precision and Regenerative Medicine and Ionian Area, School of Medicine, University of Bari “Aldo Moro”, Bari, Italy; 10 CNR-IFC, Institute of Clinical Physiology of Reggio Calabria, Reggio Calabria, Italy; 11 CNR-IFC, Institute of Clinical Physiology of Rome, Rome, Italy

**Correction: Critical Care (2024) 28:279 ** 10.1186/s13054-024-05068-x

Following publication of the original article [1], the authors identified an error in Tables 1–4. There were values missing in Table 3 and alignment/indention errors in Tables 1–4. Both the incorrect and correct tables are given hereafter.

The incorrect Table 1:

**Table 1:** Patients’ characteristics at V–V ECMO connection

	Overall population (N = 279, 100%)	Predetected patients ^(1)^ (N = 59, 21%)	V-V ECMO-acquired MDR GN ^(2)^ (N = 80, 29%)	Non-MDR GN ^(3)^ (N = 140, 50%)	*P-value*
*Baseline characteristics*					
Age, years	54 [44–61]	49 [38–58]	56 [46–62]	55 [46–62]	0.046^b^
Gender (male), n (%)	200 (72)	35 (59)	64 (80)	101 (72)	0.028^c^
IBW, Kg	65 ± 8	64 ± 10	66 ± 7	65 ± 9	0.734
Charlson Comorbidity Index (w/o age)	1 [0–2]	1 [0–2]	1 [0–1]	1 [0–2]	0.350
*Sepsis Organ Failure Assessment*					
at ICU admission	8 [6–11]	8 [6–12]	8 [7–10]	8 [5–12]	0.732
at V-V ECMO connection	9 [7–12]	10 [8–14]	9 [7–11]	8 [6–12]	0.057
IMV prior to V-V ECMO connection, days	2 [1–5]	2 [0–6]	3 [1–6]	2 [1–5]	0.067
Driving pressure at V-V ECMO initiation, cmH_2_O	16 [10–17]	15 [10–16]	15 [9–16]	15 [10–17]	0.140
PaO_2_/FiO_2_ ratio at V-V ECMO initiation	87 [67–118]	77 [59–106]	85 [72–110]	96 [79–120]	0.064
Time between H admission and V-V ECMO connection, days	4 [2–8]	4 [2–10]	4 [2–8]	4 [2–8]	0.983
Time between ICU admission and V-V ECMO connection, days	1 [0–3]	1 [0–3]	1 [0–4]	0 [0–3]	0.019^d^
*Indications for V-V ECMO support*					
Acute respiratory distress syndrome, n (%)	233 (84)	50 (85)	66 (82)	$$\left. {\begin{array}{*{20}c} {117 \, \left( {84} \right)} \\ {23 \, \left( {16} \right)} \\ \end{array} } \right\}$$	0.939
Trauma, major burn, autoimmune disease, CLAD, n (%)	46 (16)	9 (15)	14 (18)		
Interfacility transport on V-V ECMO, n (%)	79 (28)	9 (15)	33 (41)	37 (26)	0.003^e^
*Year of V-V ECMO connection, n (%)*					
2017–2019	101 (36)	16 (27)	16 (20)	$$\left. {\begin{array}{*{20}c} {69 \, \left( {49} \right)} \\ {71 \, \left( {51} \right)} \\ \end{array} } \right\}$$	< 0.001^f^
2020–2022	178 (64)	43 (73)	64 (80)		
Annual hospital V-V ECMO volume^a^, n	12 [10–20]	12 [6–12]	10 [7–20]	12 [10–22]	< 0.001^ g^

Data are presented as absolute frequency (% of the included patients) or as median and [interquartile range] or as mean ± SD. 'Predetected' group includes patients, infected or colonized, by MDR GN bacteria cultured before VV-ECMO placement

^a^Annual hospital V-V ECMO volume is defined as the specific number of patients treated with V-V ECMO per year [27]

^b^(1) vs (2) p-value 0.041, (1) vs (3) p-value 0.017

^c^(1) vs (2) p-value 0.013

^d^(2) vs (3) p-value 0.011

^e^(1) vs (2) p-value 0.001, (2) vs (3) p-value 0.025

^f^(1) vs (3) p-value 0.005, (2) vs (3) p-value < 0.001

^g^(1) vs (3) and (2) vs (3) p-values < 0.001

*ICU* Intensive Care Unit; *IMV* Invasive mechanical ventilation; *IBW* Ideal body weight; *ECMO* Extracorporeal membrane oxygenation; *MDR* Multidrug resistant; *GN* Gram-negative; *N or n* Number; *SD* Standard deviation; *w/o* Without; *V-V* Veno-venous; *CLAD* Chronic lung allograft dysfunction; *PaO*_*2*_*/FiO*_*2*_ The ratio of arterial oxygen partial pressure to fractional inspired oxygen

< 0.001^f^ referes to both lines (2017-19 and 2020-2022)

The correct Table 1:

**Table 1:**
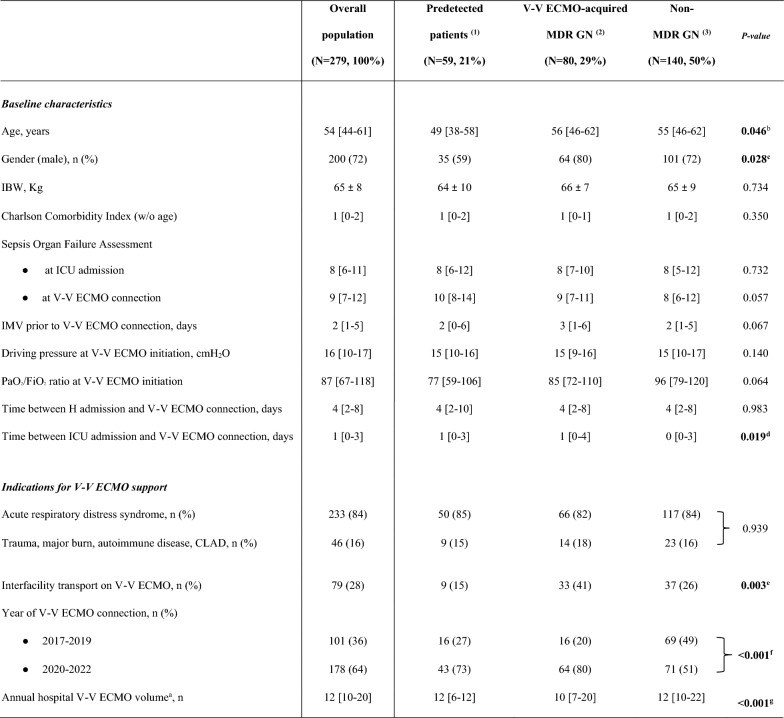
Patients’ characteristics at V-V ECMO connection.

The bold font was used for significant* p*-values.

Data are presented as absolute frequency (% of the included patients) or as median and [interquartile range] or as mean ± SD. 'Predetected' group includes patients, infected or colonized, by MDR GN bacteria cultured before VV-ECMO placement.

^a^Annual hospital V-V ECMO volume is defined as the specific number of patients treated with V-V ECMO per year [27]

^b^(1) vs (2)* p*-value 0.041, (1) vs (3) * p*-value 0.017

^c^(1) vs (2) * p*-value 0.013

^d^(2) vs (3) * p*-value 0.011

^e^(1) vs (2) * p*-value 0.001, (2) vs (3) * p*-value 0.025

^f^(1) vs (3) * p*-value 0.005, (2) vs (3) * p*-value < 0.001

^g^(1) vs (3) and (2) vs (3) * p*-values < 0.001

*ICU* Intensive Care Unit; *IMV* Invasive mechanical ventilation; *IBW* Ideal body weight; *ECMO* Extracorporeal membrane oxygenation; *MDR* Multidrug resistant; *GN* Gram-negative; *N or n* Number; *SD* Standard deviation; *w/o* Without; *V-V* Veno-venous; *CLAD* Chronic lung allograft dysfunction; *PaO*_*2*_*/FiO*_*2*_ The ratio of arterial oxygen partial pressure to fractional inspired oxygen

< 0.001^f^ referes to both lines (2017-19 and 2020-2022)

The incorrect Table 2:

**Table 2:** Outcomes

	Overall population (N = 279, 100%)	Predetected patients ^(1)^ (N = 59, 21%)	V-V ECMO-acquired MDR GN ^(2)^ (N = 80, 29%)	Non-MDR GN ^(3)^ (N = 140, 50%)	*P-value*
1-year mortality, n (%)	116 (42)	36 (61)	35 (44)	45 (32)	< 0.001^a^
infections due to MDR GN bacteria, n (%)	–	33 (56)^*^	29 (36)	–	
colonizations due to MDR GN bacteria colonization, n (%)	–	3 (5)	6 (8)	–	
Overall V-V ECMO duration, days	12 [8–22]	13 [7–28]	16 [12–26, 28]	11 [6–17]	< 0.001^b^
28-day ventilator-free days	0 [0–8]	0 [0–4]	0 [0–2]	0 [0–12]	< 0.001^c^
Weaning success, n (%)	95 (34)	17 (29)	14 (18)	$$\left. {\begin{array}{*{20}c} {64 \, \left( {45} \right)} \\ {50 \, \left( {36} \right)} \\ {26 \, \left( {19} \right)} \\ \end{array} } \right\}$$	< 0.001^d^
Weaning failure, n (%)	119 (43)	22 (37)	47 (59)		
Never extubated, n (%)	65 (23)	20 (34)	19 (24)		
RRT after V-V ECMO connection, n (%)	99 (35)	25 (42)	30 (38)	44 (31)	0.306
ICU LOS, days	27 [18–43]	22 [15–37]	39 [26–57]	24 [17–35]	< 0.001^e^

Data are presented as absolute frequency (% of the included patients) or as median and [interquartile range]

^*^Of those non-survivors, 10 subjects were pre-infected by MDR GN bacteria at V-V ECMO initiation

^a^(1) vs (3) *p* value < 0.001

^b^(1) vs (2) *p* value < 0.001, (1) vs (3) *p* value 0.043, (2) vs (3) *p* value < 0.001

^c^(1) vs (3) *p* value 0.005, (2) vs (3) *p* value < 0.001

^d^(1) vs (2) *p* value 0.042, (1) vs (3) 0.028, (2) vs (3) *p* value < 0.001

^e^(1) vs (2) *p* value < 0.001, (2) vs (3) *p* value < 0.001

*ICU* Intensive Care Unit; *RRT* Renal replacement therapy; *IMV* Invasive mechanical ventilation; *ECMO* Extracorporeal membrane oxygenation; *MDR* Multidrug resistant; *GN* Gram-negative; *N or n* Number; *V-V* Veno-venous

< 0.001^a^ referes only to the first line (1-year mortality, n (%)

The correct Table 2:

**Table 2:**
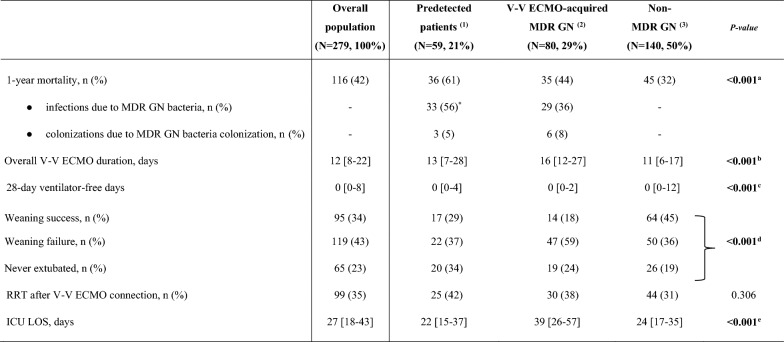
Outcomes.

The bold font was used for significant* p*-values.

Data are presented as absolute frequency (% of the included patients) or as median and [interquartile range]

*Of those non-survivors, 10 subjects were pre-infected by MDR GN bacteria at V-V ECMO initiation

^a^(1) vs (3) *p* value < 0.001

^b^(1) vs (2) *p* value < 0.001, (1) vs (3) *p* value 0.043, (2) vs (3) *p* value < 0.001

^c^(1) vs (3) *p* value 0.005, (2) vs (3) *p* value < 0.001

^d^(1) vs (2) *p* value 0.042, (1) vs (3) 0.028, (2) vs (3) *p* value < 0.001

^e^(1) vs (2) *p* value < 0.001, (2) vs (3) *p* value < 0.001

*ICU* Intensive Care Unit; *RRT* Renal replacement therapy; *IMV* Invasive mechanical ventilation; *ECMO* Extracorporeal membrane oxygenation; *MDR* Multidrug resistant; *GN* Gram-negative; *N or n* Number; *V-V* Veno-venous

< 0.001^a^ referes only to the first line (1-year mortality, n (%)

The incorrect Table 3:

**Table 3:** Microbiological characteristics of MDR GN bacteria

	Predetected patients (N = 59, 100%)	V-V ECMO-acquired MDR GN (N = 80, 100%)	*P value*
*Microbiological pattern*			
ESBL, AmpC, n (%)	10 (17)	$$\left. {\begin{array}{*{20}c} {15 \, \left( {19} \right)} \\ {65 \, \left( {81} \right)} \\ \end{array} } \right\}$$	0.960
CRE, CRAB, DTR, n (%)	49 (83)		
Infections due to MDR GN bacteria, n (%)	48 (81)^a^	$$\left. {\begin{array}{*{20}c} {61 \, \left( {76} \right)} \\ {19 \, \left( {24} \right)} \\ \end{array} } \right\}$$	0.535
Colonizations due to MDR GN bacteria, n (%)	11 (19)		
*Type of infection due to MDR GN bacteria*^*a*^			
VAP/non-VAP, n (%)	38 (64)		0.699
BSI/CR-BSI, n (%)	4 (7)^b^		
UTI, n (%)	0 (0)		
Others (i.e., soft tissue etc.), n (%)	6 (10)		

Data are presented as absolute frequency (% of the included patients)

^a^Of those patients, only 10 subjects were pre-infected by MDR GN bacteria at V-V ECMO initiation

^b^1 CR-BSI; ^c^: 2 CR-BSI. Additional information is reported in Fig. 1***.*** For more details about microbiological surveillance and diagnostic criteria see Methods and additional-Methods 1

*ECMO* extracorporeal membrane oxygenation; *MDR* Multidrug resistant; *GN* Gram-negative; *N or n* Number; *ESBL* Extended spectrum beta-lactamase; *V-V* Veno-venous; *AmpC* AmpC β-lactamase-producing; *CRE* Carbapenem-resistant Enterobacteriaceae; *DTR* Difficult-to-treat resistance (mainly Pseudomonas aeruginosa); *CRAB* Carbapenem-resistant Acinetobacter baumannii; *BSI* Blood stream infection; *VAP* Ventilator-associated pneumonia; *CR-BSI* Catheter-related bloodstream infection; *UTI* Urinary tract infection

0.960 referes to the first (ESBL, AmpC) and second line (CRE, CRAB, DTR)

The correct Table 3:

**Table 3:**
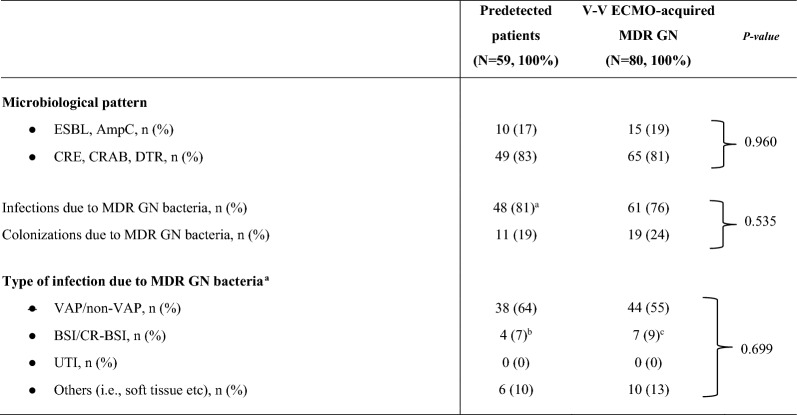
Microbiological characteristics of MDR GN bacteria.

Data are presented as absolute frequency (% of the included patients)

^a^Of those patients, only 10 subjects were pre-infected by MDR GN bacteria at V-V ECMO initiation

^b^1 CR-BSI

^c^: 2 CR-BSI. Additional information is reported in Fig. 1***.*** For more details about microbiological surveillance and diagnostic criteria see Methods and additional-Methods 1

*ECMO* extracorporeal membrane oxygenation; *MDR* Multidrug resistant; *GN* Gram-negative; *N or n* Number; *ESBL* Extended spectrum beta-lactamase; *V-V* Veno-venous; *AmpC* AmpC β-lactamase-producing; *CRE* Carbapenem-resistant Enterobacteriaceae; *DTR* Difficult-to-treat resistance (mainly Pseudomonas aeruginosa); *CRAB* Carbapenem-resistant Acinetobacter baumannii; *BSI* Blood stream infection; *VAP* Ventilator-associated pneumonia; *CR-BSI* Catheter-related bloodstream infection; *UTI* Urinary tract infection

0.960 referes to the first (ESBL, AmpC) and second line (CRE, CRAB, DTR)

The incorrect Table 4:

**Table 4:** Concomitant pathogens and antibiotics.

	Overall population (N = 279, 100%)	Predetected patients (N = 59, 21%)	V-V ECMO-acquired MDR GN (N = 80, 29%)	Non-MDR GN^*^ (N = 140, 50%)	*P-value*
*Concomitant isolation of*					
Sars-Cov-2, influenza virus, n (%)	157 (56)	34 (58)	40 (50)	83 (59)	0.399
Candida sp., n (%)	73 (26)	13 (22)	24 (30)	36 (26)	0.564
Aspergillus sp., n (%)	41 (15)	7 (12)^c^	17 (21)^d^	17 (12)^e^	0.146
Concomitant infections due to Gram-positive bacteria^a^, n (%)	103 (37)	20 (34)	31 (39)	52 (37)	0.840
*Resistance pattern*^*b*^* (only Gram-positive bacteria)*					
VRE, n (%)	29 (10)	8 (14)	10 (13)	$$\left. {\begin{array}{*{20}c} {11 \, \left( 8 \right)} \\ {46 \, \left( {33} \right)} \\ {13 \, \left( 9 \right)} \\ \end{array} } \right\}$$	0.645
Multi-sensitive, n (%)	91 (33)	17 (29)	28 (35)		
Other resistances (i.e. LRE), n (%)	32 (11)	9 (15)	10 (13)		
*Empiric broad-spectrum antibiotics*					
Penicillins, β-lactam-inhibitor/III, IV cephalosporins or fluoroquinolones, n (%)	132 (47)	27 (46)	39 (49)	$$\left. {\begin{array}{*{20}c} {66 \, \left( {47} \right)} \\ {44 \, \left( {32} \right)} \\ {30 \, \left( {21} \right)} \\ \end{array} } \right\}$$	0.915
Carbapenems, ceftazidime-avibactam, ceftolozane-tazobactam, cefiderocol, etc., n (%)	82 (29)	17 (29)	21 (26)		
Only targeted therapy or nothing, n (%)	65 (24)	15 (25)	20 (25)		

Data are presented as absolute frequency (% of the included patients) or as median and [interquartile range]. For more details about microbiological surveillance see Methods and additional-Methods 1

^*^Moreover, 39 (28%) subjects detected multisensitive GN bacteria and only 23 (16%) patients never recorded positive cultures

^a^for more details concerning Gram-positive bacteria see additional-Table 6

^b^in case of multiple bacterial isolations, only the worst resistance pattern was counted

^c^1 out of 7 patients isolated Candida sp. and Aspergillus sp. simultaneously

^d^3 out of 17 patients isolated Candida sp. and Aspergillus sp. simultaneously

^e^1 out of 7 patients isolated Candida sp. and Aspergillus sp. simultaneously

*ECMO* Extracorporeal membrane oxygenation; *MDR* Multidrug resistant; *GN* Gram-negative; *N or n* Number; *VRE* Vanco-resistant enterococcus; *LRE* Linezolid-resistant enterococcus; *V-V* Veno-venous; *sp* Species

The correct Table 4:

**Table 4:**
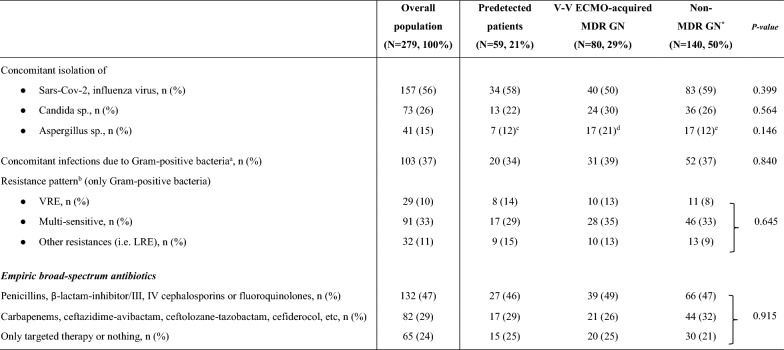
Concomitant pathogens and antibiotics.

Data are presented as absolute frequency (% of the included patients) or as median and [interquartile range]. For more details about microbiological surveillance see Methods and additional-Methods 1.

*Moreover, 39 (28%) subjects detected multisensitive GN bacteria and only 23 (16%) patients never recorded positive cultures.

^a^for more details concerning Gram-positive bacteria see additional-Table 6.

^b^in case of multiple bacterial isolations, only the worst resistance pattern was counted.

^c^1 out of 7 patients isolated Candida sp. and Aspergillus sp. simultaneously.

^d^3 out of 17 patients isolated Candida sp. and Aspergillus sp. simultaneously.

^e^1 out of 7 patients isolated Candida sp. and Aspergillus sp. simultaneously.

*ECMO* Extracorporeal membrane oxygenation; *MDR* Multidrug resistant; *GN* Gram-negative; *N or n* Number; *VRE* Vanco-resistant enterococcus; *LRE* Linezolid-resistant enterococcus; *V-V* Veno-venous; *sp* Species.

Tables 1, 2, 3 and 4 have been updated in this correction article and the original article [1] has been corrected.
