# Supplementary material for: Retrospective ANalysis of multi-drug resistant Gram-nEgative bacteRia on veno-venous extracorporeal membrane oxygenation. The multicenter RANGER STUDY
Source: Crit Care. 2024 Aug 27;28:279. doi: 10.1186/s13054-024-05068-x (PMC11351604; doi:10.1186/s13054-024-05068-x)
Supplement: Supplementary file 1 — Additional file1 [file 13054_2024_5068_MOESM1_ESM.docx]

**ADDITIONAL FILES**

**ARTICLE TITLE: Retrospective ANalysis of multi-drug resistant Gram-nEgative bacteRia on veno-venous extracorporeal membrane oxygenation. The multicenter RANGER STUDY.**

**Additional-Table 1. STROBE Statement—Checklist.**

**additional-Methods 1. Definitions and protocols.**

**additional-Table 2. Cox-proportional hazards model for 1-year mortality (multivariable analysis between three predefined groups).**

**additional-Table 3. Additional data on 1-year mortality (multivariable analysis between predetected patients vs ‘V-V ECMO-acquired MDR GN bacteria’ group).**

**additional-Table 4 Cox-proportional hazards model for 1-year mortality (univariable analysis).**

**additional-Table 5. Univariable and multivariable logistic regression model for the (unadjusted and adjusted) risk of MDR GN bacteria occurrence according to annual hospital V-V ECMO volume.**

**additional-Figure 1. Flow-chart.**

**addtional-Table 6. Overall GP bacteria: site of isolation.**

**References.**

**Additional-Table 1. STROBE Statement—Checklist^1^.**

|  | **Item No** | **Recommendation** | **Page No** |
| --- | --- | --- | --- |
| **Title and abstract** | 1 | (*a*) Indicate the study’s design with a commonly used term in the title or the abstract | 1, 4-5 |
|  |  | (*b*) Provide in the abstract an informative and balanced summary of what was done and what was found |  |
| **Introduction** | | | |
| Background/rationale | 2 | Explain the scientific background and rationale for the investigation being reported | 5 |
| Objectives | 3 | State specific objectives, including any prespecified hypotheses | 6 |
| **Methods** | | | |
| Study design | 4 | Present key elements of study design early in the paper | 7, 8 |
| Setting | 5 | Describe the setting, locations, and relevant dates, including periods of recruitment, exposure, follow-up, and data collection | 7, 8 |
| Participants | 6 | (*a*) Give the eligibility criteria, and the sources and methods of selection of participants. Describe methods of follow-up | 7 |
|  |  | (*b*) For matched studies, give matching criteria and number of exposed and unexposed | -- |
| Variables | 7 | Clearly define all outcomes, exposures, predictors, potential confounders, and effect modifiers. Give diagnostic criteria, if applicable | 8, 9 |
| Data sources/ measurement | 8* | For each variable of interest, give sources of data and details of methods of assessment (measurement). Describe comparability of assessment methods if there is more than one group | 10 |
| Bias | 9 | Describe any efforts to address potential sources of bias | - |
| Study size | 10 | Explain how the study size was arrived at | 10 |
| Quantitative variables | 11 | Explain how quantitative variables were handled in the analyses. If applicable, describe which groupings were chosen and why | 10, 11 |
| Statistical methods | 12 | (*a*) Describe all statistical methods, including those used to control for confounding | 10, 11 |
|  |  | (*b*) Describe any methods used to examine subgroups and interactions |  |
|  |  | (*c*) Explain how missing data were addressed |  |
|  |  | (*d*) If applicable, explain how loss to follow-up was addressed | - |
|  |  | (*e*) Describe any sensitivity analyses | - |
| **Results** | | |  |
| Participants | 13* | (a) Report numbers of individuals at each stage of study—eg numbers potentially eligible, examined for eligibility, confirmed eligible, included in the study, completing follow-up, and analysed | 11 |
|  |  | (b) Give reasons for non-participation at each stage |  |
|  |  | (c) Consider use of a flow diagram |  |
| Descriptive data | 14* | (a) Give characteristics of study participants (eg demographic, clinical, social) and information on exposures and potential confounders | 11-12 |
|  |  | (b) Indicate number of participants with missing data for each variable of interest | - |
|  |  | (c) Summarise follow-up time (eg, average and total amount) | - |
| Outcome data | 15* | Report numbers of outcome events or summary measures over time | 11, 12 |
| Main results | 16 | (a) Give unadjusted estimates and, if applicable, confounder-adjusted estimates and their precision (eg, 95% confidence interval). Make clear which confounders were adjusted for and why they were included  (b) Report category boundaries when continuous variables were categorized  (c) If relevant, consider translating estimates of relative risk into absolute risk for a meaningful time period | 11-13 |
| Other analyses | 17 | Report other analyses done—eg analyses of subgroups and interactions, and sensitivity analyses | - |
| **Discussion** |  |  |  |
| Key results | 18 | Summarise key results with reference to study objectives | 13-15 |
| Limitations | 19 | Discuss limitations of the study, taking into account sources of potential bias or imprecision.  Discuss both direction and magnitude of any potential bias | 16 |
| Interpretation | 20 | Give a cautious overall interpretation of results considering objectives, limitations, multiplicity of analyses, results from similar studies, and other relevant evidence | 17 |
| Generalisability | 21 | Discuss the generalisability (external validity) of the study results | - |
| **Other information** |  |  |  |
| Funding | 22 | Give the source of funding and the role of the funders for the present study and, if applicable, for the original study on which the present article is based | 20, 21 |

**Additional-Methods 1. Definitions and protocols.**

All infections were classified according to the CDC/NHSN criteria (last released manual available on <https://www.cdc.gov/nhsn/pdfs/pscmanual/17pscnosinfdef_current.pdf>), as follows: ventilator-associated pneumonia (VAP) (occurring at least 48 hours after intubation, otherwise we made diagnosis of non-VAP), bloodstream infection (BSI)/catheter-related bloodstream infection (CR-BSI) and urinary tract infection (UTI) (at least 48 hours after catheterization)^2-8^. Soft tissue infections and other forms of infections were named as ‘Others’^2-8^. In the case of pre-detected MDROs, patients developing infections from an initial pre-colonizations were listed as infected in ***Tables 2*** and ***3***.

Diagnostic criteria according to the infection site are listed above:

| **Infection** | **Site of Culture** | **Bacterial Load** | **Clinical Signs** | **Also** |
| --- | --- | --- | --- | --- |
| **Blood Stream Infection^a^** | 2 or more blood specimens collected on separte occasions | --- | At least one of the following signs or symptoms: fever (>38.0^o^C), chills, or hypotension | No further sign of localized infection.  A laboratory confirmed bloodstream infection  where an eligible BSI organism is identified. |
| **Catheter-related ^a^** or **central line-associated blood stream infection** | 2 or more blood specimens collected on separte occasions | --- | Patient of any age has at least one of the  following signs or symptoms: fever  (>38.0^°^C), chills, or hypotension.  Eventual erythema, swelling, purulent drainage  from catheter insertion-site. | No further sign of localized infection.  A laboratory confirmed CR-bloodstream infection where an eligible BSI organism is identified, and an eligible central line is present on the Laboratory Confirmed Bloodstream Infection  date of event or the day before. |
| **Ventilator-Associated Pneumonia^b*^** | Bronchoalveolar lavage | ≥ 10^4^ CFU/mL | Signs and symptoms:  for any patient, at least one of the following:  - fever (>38°c)  - leukopenia (<=4000 wbc/mm3) or  leukocytosis (>=12000 wbc/mm3)  - for adults >=70 years old, altered mental status  with no other recognized cause  and  at least two of the following (from separate  bullets):  - new onset of purulent sputum or change in  character of sputum, or increased respiratory  secretions, or increased suctioning requirements  - dyspnea or tachypnea or new onset or  worsening cough  - rales or bronchial breath sounds  - worsening gas exchange (for example, O_2_  desaturation, increased oxygen requirements, or  increased ventilator demand) | Imaging:  two or more serial chest imaging test result with  one of the following:  new and persistent  or  progressive and persistent  - infiltrate  - consolidation  - cavitation |
|  | Endotracheal Aspirate | ≥ 10^5^ CFU/mL |  |  |
| **Urinary Tract Infection^c^** | 2 consecutive urine  specimens^4^ | ≥ 10^5^ CFU/mL | At least one of the following signs or symptoms:  - fever  - suprapubic tenderness  - costovertebral angle pain or tenderness  - urinary urgency (when catheter not in place)  - urinary frequency (when catheter not in place)  - dysuria (when catheter not in place) | |

Diagnostic criteria inspired by literature^2-8^. ^a^: <https://www.cdc.gov/nhsn/pdfs/pscmanual/4psc_clabscurrent.pdf>; ^b^: <https://www.cdc.gov/nhsn/pdfs/pscmanual/6pscvapcurrent.pdf> ; ^c^: <https://www.cdc.gov/nhsn/pdfs/pscmanual/7psccauticurrent.pdf> . For more information about other infections <https://www.cdc.gov/nhsn/pdfs/pscmanual/17pscnosinfdef_current.pdf>. Infections due to Clostridium Difficile were not found. *Abbreviations:* CFU, colony forming units.

Moreover, in all enrolled hospitals, a standardized protocol for infection control and prevention was applied, as follows: i) all the positive microbial cultures were independently evaluated, considering the available clinical, laboratory and radiographic data, by specialized intensivists and infectious diseases specialists; ii) antibiotic regimens were revised daily with dedicated infectious diseases specialist consultants and a daily communication with the microbiology laboratory; iii) no prophylactic antibiotic was provided at V-V ECMO cannulation; iv) all patients were maintained in semi-recumbent position for preventing inhalation; v) systematic stress ulcer prophylaxis was administered daily; vi) early enteral feeding was encouraged; vii) alcohol based hand hygiene and daily whole body bathing were strongly recommended^8-10^. Finally, strict individual contact precautions and patient cohort isolation were applied to all patients with MDR GN bacteria isolations^9^.

**Additional-Table 2. Cox-proportional hazards model for 1-year mortality (multivariable analysis between three predefined groups).**

| **Multivariable analysis for 1-year mortality** | | | |
| --- | --- | --- | --- |
| **Characteristics** | **aHR** | **95% CI** | ***P-value*** |
|  |  |  |  |
| Predetected patients | **2.14** | **1.33 – 3.47** | **0.002** |
| V-V ECMO-acquired MDR GN | 1.51 | 0.94 – 2.42 | 0.090 |
| Non-MDR GN (reference) | 1.00 | - | - |
| Age, years | **1.03** | **1.01 – 1.04** | **0.006** |
| Sepsis Organ Failure Assessment at V-V ECMO connection | **1.06** | **1.01 – 1.12** | **0.033** |
| Interfacility transport on V-V ECMO (reference: no) | **0.57** | **0.34 – 0.94** | **0.027** |
| Annual hospital V-V ECMO volume, n | 0.96 | 0.91 – 1.01 | 0.150 |
| **Multivariable analysis for 1-year mortality**  ***(considering only patients with infections due to MDR GN bacteria)** | | | |
| **Characteristics** | **aHR** | **95% CI** | ***P-value*** |
| Predetected patients* | **2.35** | **1.44 – 3.86** | **< 0.001** |
| V-V ECMO-acquired MDR GN* | 1.57 | 0.95 – 2.57 | 0.076 |
| Non-MDR GN (reference) | 1.00 | - | - |
| Age, years | **1.02** | **1.01 – 1.04** | **0.012** |
| Sepsis Organ Failure Assessment at V-V ECMO connection | 1.04 | 0.99 – 1.10 | 0.147 |
| Interfacility transport on V-V ECMO (reference: no) | 0.65 | 0.39 – 1.10 | 0.111 |
| Annual hospital V-V ECMO volume, n | 0.97 | 0.93 – 1.01 | 0.103 |
| **Multivariable analysis for 1-year mortality**  ****(considering only patients with predetected MDR GN bacteria)** | | | |
| **Characteristics** | **aHR** | **95% CI** | ***P-value*** |
| Pre-infected patients** | **4.44** | **1.69 – 11.66** | **0.002** |
| Pre-colonized patients** | **2.25** | **1.02 – 4.98** | **0.044** |
| Non-MDR GN (reference) | 1.00 | - | - |
| Age, years | **1.03** | **1.01 – 1.06** | **0.020** |
| Sepsis Organ Failure Assessment at V-V ECMO connection | 1.05 | 0.97 – 1.14 | 0.196 |
| Interfacility transport on V-V ECMO (reference: no) | **0.30** | **0.12 – 0.74** | **0.009** |
| Annual hospital V-V ECMO volume, n | **0.99** | **0.93 – 1.06** | **0.005** |

Data are presented as aHR and [95% CI]. The adjustment was provided according to the significant variables (p < 0.05) identified in two univariable models reported in ***additional-Table 4***. *Abbreviations:* ECMO: extracorporeal membrane oxygenation; MDR: multidrug resistant; GN: Gram-negative; CI: confidential interval; aHR: adjusted hazard ratio; n: number.

**Additional-Table 3. Additional data on 1-year mortality (multivariable analysis between predetected patients vs ‘V-V ECMO-acquired MDR GN bacteria’ group).**

| **Multivariable analysis for 1-year mortality** | | | |
| --- | --- | --- | --- |
| **Characteristics** | **aHR** | **95% CI** | ***P-value*** |
|  |  |  |  |
| Predetected patients | 1.42 | 0.86 – 2.36 | 0.173 |
| V-V ECMO-acquired MDR GN (reference) | 1.00 | - | - |
| Age, years | 1.02 | 1.00 – 1.04 | 0.086 |
| Sepsis Organ Failure Assessment at V-V ECMO connection | 1.07 | 0.99 – 1.15 | 0.055 |
| Interfacility transport on V-V ECMO (reference: no) | 0.75 | 0.40 – 1.39 | 0.360 |
| Annual hospital V-V ECMO volume, n | 0.99 | 0.94 – 1.03 | 0.550 |

Data are presented as aHR and [95% CI]*.* The adjustment was provided according to the significant variables (p < 0.05) identified in two univariable models reported in ***additional-Table 4***. *Abbreviations:* ECMO: extracorporeal membrane oxygenation; MDR: multidrug resistant; GN: Gram-negative; CI: confidential interval; V-V: veno-venous; aHR: adjusted hazard ratio; n: number.

**Additional-Table 4 Cox-proportional hazards model for 1-year mortality (univariable analysis).**

| **Univariable analysis for 1-year mortality** | | | | |
| --- | --- | --- | --- | --- |
| **Characteristics** | **Proportional-hazard assumption** | **HR** | **95% CI** | ***P-value*** |
|  |  |  |  |  |
| Predetected patients | 0.63 | **2.30** | **1.48 – 3.56** | **< 0.001** |
| V-V ECMO-acquired MDR GN | - | 1.49 | 0.96 – 2.32 | 0.076 |
| Non-MDR GN (reference) | - | 1.00 | - | - |
| Age, years | 0.31 | **1.02** | **1.01 – 1.04** | **0.010** |
| Gender (reference: male) | 0.53 | 0.82 | 0.54 – 1.25 | 0.353 |
| Sepsis Organ Failure Assessment at V-V ECMO connection | 0.16 | **1.09** | **1.04 – 1.15** | **< 0.001** |
| IMV prior to V-V ECMO connection, days | 0.10 | 1.01 | 0.99 – 1.03 | 0.218 |
| PaO_2_/FiO_2_ ratio at V-V ECMO initiation | 0.17 | 1.00 | 0.99 – 1.01 | 0.874 |
| Time between ICU admission and V-V ECMO start, days | 0.73 | 1.01 | 0.99 – 1.03 | 0.311 |
| Interfacility transport on V-V ECMO (reference: no) | 0.21 | **0.499** | **0.31 – 0.80** | **0.004** |
| Annual hospital V-V ECMO volume, n | 0.32 | **0.96** | **0.93 – 0.99** | **0.007** |
| Year of V-V ECMO connection (reference: 2017-2019) | 0.64 | 1.41 | 0.95 – 2.09 | 0.091 |
| **Univariable analysis for 1-year mortality**  ***(considering only patients with infections due to MDR GN bacteria)** | | | | |
| **Characteristics** | **Proportional-hazard assumption** | **HR** | **95% CI** | ***P-value*** |
|  |  |  |  |  |
| Predetected patients* | 0.24 | **9.15** | **4.13 – 20.27** | **< 0.001** |
| V-V ECMO-acquired MDR GN* | - | **2.88** | **1.58 – 5.26** | **< 0.001** |
| Non-MDR GN (reference) | - | 1.00 | - | - |
| **Univariable analysis for 1-year mortality**  ****(considering only patients with predetected MDR GN bacteria)** | | | | |
| **Characteristics** | **Proportional-hazard assumption** | **HR** | **95% CI** | ***P-value*** |
|  |  |  |  |  |
| Pre-infected patients** | 0.35 | **2.72** | **1.74 – 4.28** | **< 0.001** |
| Pre-colonized patients** | - | **1.68** | **1.05 – 2.67** | **0.030** |
| Non-MDR GN (reference) | - | 1.00 | - | - |

Data are presented as HR and [95% CI]. *Abbreviations:* ECMO: extracorporeal membrane oxygenation; MDR: multidrug resistant; IMV: invasive mechanical ventilation; ICU: intensive care unit; GN: Gram-negative; CI: confidential interval; V-V: veno-venous; HR: hazard ratio; n: number; PaO_2_/FiO_2_: the ratio of arterial oxygen partial pressure to fractional inspired oxygen.

**Additional-Table 5. Univariable and multivariable logistic regression model for the (unadjusted and adjusted) risk of MDR GN bacteria occurrence according to annual hospital V-V ECMO volume.**

| **Univariable analysis for the risk of V-V ECMO-acquired MDR GN bacteria** | | | |
| --- | --- | --- | --- |
| **Characteristics** | **OR** | **95% CI** | ***P-value*** |
| Annual hospital V-V ECMO volume, n | **0.91** | **0.86 – 0.96** | **< 0.001** |
| Age, years | 0.99 | 0.97 – 1.02 | 0.735 |
| Gender (reference: male) | 1.11 | 0.61 – 2.03 | 0.735 |
| Sepsis Organ Failure Assessment at V-V ECMO connection | 1.00 | 0.93 – 1.08 | 0.967 |
| IMV prior to V-V ECMO connection, days | **1.07** | **1.01 – 1.13** | **0.024** |
| PaO_2_/FiO_2_ ratio at V-V ECMO initiation | 0.99 | 0.98 – 1.00 | 0.133 |
| Time between ICU admission and V-V ECMO start, days | 1.04 | 0.99 – 1.10 | 0.159 |
| Interfacility transport on V-V ECMO (reference: no) | **1.95** | **1.09 – 3.50** | **0.024** |
| Year of V-V ECMO connection (reference: 2017-2019) | **3.89** | **2.05 – 7.37** | **< 0.001** |
| **Multivariable analysis for the risk of V-V ECMO-acquired MDR GN bacteria** | | | |
| **Characteristics** | **aOR** | **95% CI** | ***P-value*** |
|  |  |  |  |
| Annual hospital V-V ECMO volume, n | **0.91** | **0.86 – 0.97** | **0.002** |
| IMV prior to V-V ECMO connection, days | 1.06 | 1.00 – 1.13 | 0.065 |
| Interfacility transport on V-V ECMO (reference: no) | **3.82** | **1.86 – 7.80** | **< 0.001** |
| Year of V-V ECMO connection (reference: 2017-2019) | **4.06** | **2.02 – 8.14** | **< 0.001** |

Data are presented as OR or aOR and [95% CI]*. Abbreviations:* ECMO: extracorporeal membrane oxygenation; MDR: multidrug resistant; IMV: invasive mechanical ventilation; GN: Gram-negative; CI: confidential interval; V-V: veno-venous; ICU: intensive care unit; OR: odds ratio; aOR: adjusted odds ratio; n: number; PaO_2_/FiO_2_: the ratio of arterial oxygen partial pressure to fractional inspired oxygen.

**Additional-Figure 1. Flow-chart.**


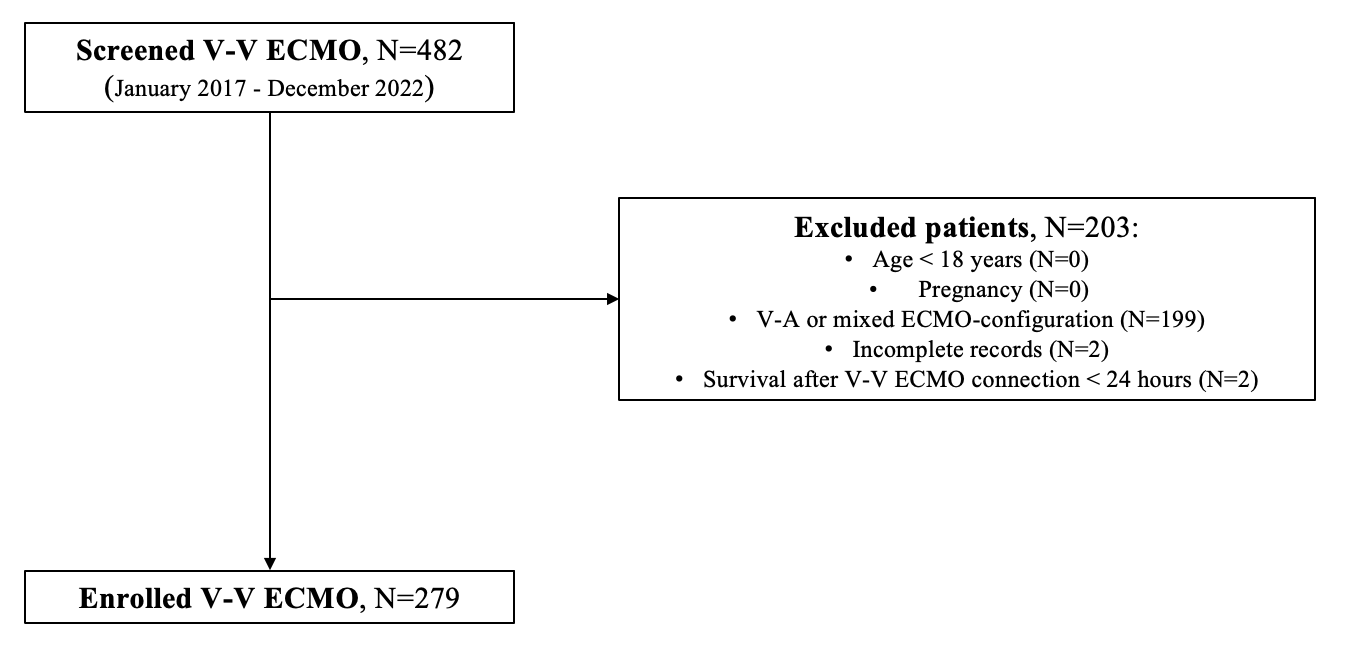


Specifically, 58 V-V ECMO patients were enrolled at Mater Domini Hospital (Catanzaro); 51 patients at Padua University Hospital; 32 at Verona University Hospital; 35 at Policlinico University Hospital (Bari); and 114 subjects at Fondazione IRCCS San Gerardo dei Tintori Hospital (Monza). *Abbreviations:* ECMO: extracorporeal membrane oxygenation; V-V: veno-venous; V-A: veno-arterial; n: number.

**Additional-Table 6. Overall GP bacteria: site of isolation.**

| **MDR GP bacteria** | | **N. of patients for site** | | | | | |
| --- | --- | --- | --- | --- | --- | --- | --- |
|  |  |  | ***Blood stream*** | ***Respiratory tract*** | ***Urinary tract*** | ***Rectal swab*** | ***Other*** |
|  |  |  |  |  |  |  |  |
|  | *Enterococcus faecalis* |  | 16 (6) | 2 (1) | 6 (2) | - | 3 (1) |
|  | *Enterococcus faecium* |  | 17 (6) | 4 (2) | 10 (4) | 14 (5) | - |
|  | *Staphylococcus epidermidis* |  | 22 (8) | 1 (1) | 1 (1) | 1 (1) | 2 (1) |
|  | *Staphylococcus aureus* |  | 8 (3) | 39 (14) | - | - | 1 (1) |
|  | *Staphylococcus hominis, haemolyticus* |  | 14 (5) | - | - | - | - |
|  | *Others^a^* |  | 5 (2) | 10 (4) | 8 (3) | - | - |

Values are expressed as number of patients with GP bacteria isolation or as (percentage) of the overall population (n. 279). For more details about microbiological surveillance see ***Methods*** and ***addtional-Methods 1***. ^a^: Streptococcus pneumoniae, pyogenes, parasanguinis etc. *Abbreviations:* GP: Gram-positive; N: number.

**REEFERENCES**

1. von Elm E, Altman DG, Egger M, Pocock SJ, Gøtzsche PC, Vandenbroucke JP, et al. The Strengthening the Reporting of Observational Studies in Epidemiology (STROBE) statement: guidelines for reporting observational studies. PLoS Med. 2007 Oct 16;4(10):e296.

2. Hooton TM, Bradley SF, Cardenas DD, et al. Diagnosis, prevention, and treatment of catheter-associated urinary tract infection in adults: 2009 International Clinical Practice Guidelines from the Infectious Diseases Society of America. *Clin Infect Dis*. 2010;50(5):625-663.

3. Mermel LA, Allon M, Bouza E, et al. Clinical practice guidelines for the diagnosis and management of intravascular catheter-related infection: 2009 Update by the Infectious Diseases Society of America. *Clin Infect Dis*. 2009;49(1):1-45.

4. [Horan](https://pubmed.ncbi.nlm.nih.gov/?term=Horan+TC&cauthor_id=18538699) TC, [Andrus](https://pubmed.ncbi.nlm.nih.gov/?term=Andrus+M&cauthor_id=18538699) M, [Dudeck](https://pubmed.ncbi.nlm.nih.gov/?term=Dudeck+MA&cauthor_id=18538699) MA. CDC/NHSN surveillance definition of health care-associated infection and criteria for specific types of infections in the acute care setting. Am J Infect Control. 2008 Jun;36(5):309-32.

5. American Thoracic Society; Infectious Diseases Society of America. Guidelines for the management of adults with hospital-acquired, ventilator-associated, and healthcare-associated pneumonia. *Am J Respir Crit Care Med*. 2005;171(4):388-416.

6. Tamma PD, Aitken SL, Bonomo RA, Mathers AJ, van Duin D, Clancy CJ. Infectious Diseases Society of America 2023 Guidance on the Treatment of Antimicrobial Resistant Gram-Negative Infections. Clin Infect Dis Off Publ Infect Dis Soc Am. 2023 Jul 18;ciad428.

7. Haneke F, Schildhauer TA, Schlebes AD, Strauch JT, Swol J. Infections and Extracorporeal Membrane Oxygenation: Incidence, Therapy, and Outcome. ASAIO J Am Soc Artif Intern Organs 1992. 2016;62(1):80–6.

8. Tonna JE, Abrams D, Brodie D, Greenwood JC, Rubio Mateo-Sidron JA, Usman A, et al. Management of Adult Patients Supported with Venovenous Extracorporeal Membrane Oxygenation (VV ECMO): Guideline from the Extracorporeal Life Support Organization (ELSO). ASAIO J Am Soc Artif Intern Organs. 2021 Jun 1;67(6):601–10.

9. Tiseo G, Brigante G, Giacobbe DR, Maraolo AE, Gona F, Falcone M, et al. Diagnosis and management of infections caused by multidrug-resistant bacteria: guideline endorsed by the Italian Society of Infection and Tropical Diseases (SIMIT), the Italian Society of Anti-Infective Therapy (SITA), the Italian Group for Antimicrobial Stewardship (GISA), the Italian Association of Clinical Microbiologists (AMCLI) and the Italian Society of Microbiology (SIM). Int J Antimicrob Agents. 2022 Aug;60(2):106611.

10. Klompas M, Branson R, Cawcutt K, Crist M, Eichenwald EC, Greene LR, et al. Strategies to prevent ventilator-associated pneumonia, ventilator-associated events, and nonventilator hospital-acquired pneumonia in acute-care hospitals: 2022 Update. Infect Control Hosp Epidemiol. 2022 Jun;43(6):687–713.
